# Identification of the initial molecular changes in response to circulating angiogenic cells-mediated therapy in critical limb ischemia

**DOI:** 10.1186/s13287-020-01591-0

**Published:** 2020-03-06

**Authors:** Lucia Beltran-Camacho, Margarita Jimenez-Palomares, Marta Rojas-Torres, Ismael Sanchez-Gomar, Antonio Rosal-Vela, Sara Eslava-Alcon, Mª Carmen Perez-Segura, Ana Serrano, Borja Antequera-González, Jose Angel Alonso-Piñero, Almudena González-Rovira, Mª Jesús Extremera-García, Manuel Rodriguez-Piñero, Rafael Moreno-Luna, Martin Røssel Larsen, Mª Carmen Durán-Ruiz

**Affiliations:** 1 Biomedicine, Biotechnology and Public Health Department, Cádiz University, Cadiz, Spain; 2 Institute of Biomedical Research Cadiz (INIBICA), Cadiz, Spain; 3 Angiology & Vascular Surgery Unit, Hospital Universitario Puerta del Mar, Cádiz, Spain; 4 Laboratory of Neuroinflammation, Hospital Nacional de Paraplejicos, SESCAM, Toledo, Spain; 5 Department of Biochemistry and Molecular Biology, University of Southern Denmark, Odense, Denmark

**Keywords:** Critical limb ischemia, Circulating angiogenic cells, Proteomics, Angiogenesis, Arteriogenesis

## Abstract

**Background:**

Critical limb ischemia (CLI) constitutes the most aggressive form of peripheral arterial occlusive disease, characterized by the blockade of arteries supplying blood to the lower extremities, significantly diminishing oxygen and nutrient supply. CLI patients usually undergo amputation of fingers, feet, or extremities, with a high risk of mortality due to associated comorbidities.

Circulating angiogenic cells (CACs), also known as early endothelial progenitor cells, constitute promising candidates for cell therapy in CLI due to their assigned vascular regenerative properties. Preclinical and clinical assays with CACs have shown promising results. A better understanding of how these cells participate in vascular regeneration would significantly help to potentiate their role in revascularization.

Herein, we analyzed the initial molecular mechanisms triggered by human CACs after being administered to a murine model of CLI, in order to understand how these cells promote angiogenesis within the ischemic tissues.

**Methods:**

Balb-c nude mice (n:24) were distributed in four different groups: healthy controls (C, n:4), shams (SH, n:4), and ischemic mice (after femoral ligation) that received either 50 μl physiological serum (SC, n:8) or 5 × 10^5^ human CACs (SE, n:8). Ischemic mice were sacrificed on days 2 and 4 (n:4/group/day), and immunohistochemistry assays and qPCR amplification of Alu-human-specific sequences were carried out for cell detection and vascular density measurements. Additionally, a label-free MS-based quantitative approach was performed to identify protein changes related.

**Results:**

Administration of CACs induced in the ischemic tissues an increase in the number of blood vessels as well as the diameter size compared to ischemic, non-treated mice, although the number of CACs decreased within time. The initial protein changes taking place in response to ischemia and more importantly, right after administration of CACs to CLI mice, are shown.

**Conclusions:**

Our results indicate that CACs migrate to the injured area; moreover, they trigger protein changes correlated with cell migration, cell death, angiogenesis, and arteriogenesis in the host. These changes indicate that CACs promote from the beginning an increase in the number of vessels as well as the development of an appropriate vascular network.

**Graphical abstract:**

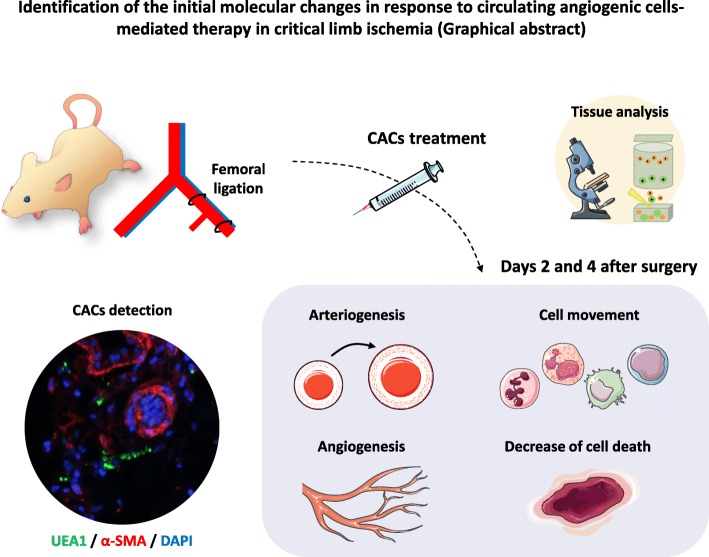

## Introduction

Critical limb ischemia (CLI) constitutes the most aggressive form of peripheral arterial obstructive disease (PAOD), a cardiovascular disease (CVD) mainly caused by atherosclerosis, resulting in blockade of arteries supplying blood to the lower extremities [1]. CLI presents as rest pain, ischemic ulceration, or foot gangrene. Patients with CLI have a high risk of limb loss and fatal or non-fatal vascular events, such as myocardial infarction (MI) and stroke [1]. This disease affects more than 202 million people around the world and up to 10–15% of the aged adult population [2]. CLI reduces significantly the quality of life of patients, which usually undergo amputation of the fingers, toes, or extremities, with an annual rate of amputation of 30% and 25% of mortality related [3–5]. In addition, metabolic alterations such as diabetes mellitus significantly increase the risk of PAOD, accelerating its course and making these patients more susceptible to ischemic events and impaired functional status compared to patients without diabetes [6].

Unfortunately, no drugs have been approved for the treatment of PAOD or CLI, and the available symptomatic treatments are ineffective [7]. Conventional surgical revascularization is not possible in 50–90% of patients due to high comorbidity [8, 9]. Moreover, approximately 30–50% of patients will require repeated interventions as treatments are often ineffective and fail within 1 year [10]. Thus, angiogenic therapy, which involves the use of an exogenous stimulus to promote blood vessel growth, is a highly promising approach for the treatment of ischemic diseases [11]. In this sense, endothelial progenitor cells (EPCs) have arisen as promising candidates for therapeutic applications pursuing tissue revascularization such as in CLI due to their assigned vascular regenerative properties [12]. Since their discovery in 1997 [13], many researchers have explored the potential of using EPCs in tissue engineering as an angiogenic source for vascular repairing [14, 15], with many publications also focused on the controversy regarding isolation techniques as well as the definition of EPC phenotypes, presenting still a variety of results in terms of surface-based EPC markers [14, 16, 17]. In this regard, a consensus has been reached at least in the need to discriminate between two different populations of the so-called EPCs, depending on the differentiation status or their capability to form colonies [17–20]: early EPCs (eEPCs), also defined as circulating angiogenic cells (CACs) or myeloid angiogenic cells (MACs), and late outgrowth EPCs or endothelial colony-forming cells (ECFCs). Both populations seem to play a significant role in the revascularization process, the first ones more likely in a paracrine fashion (not so much deriving into an endothelial phenotype) while ECFCs participate by replacing damaged endothelial cells (ECs) [15, 17].

The discovery of EPCs has promoted the development of cell-based therapies applied in ischemic cardiovascular diseases including PAOD [21]. Bone marrow (BM) or peripheral blood (PB) mononuclear cells (MNCs), including EPCs as well as EPC-enriched fractions, have been preclinically applied with promising results, encouraging the design of clinical trials [21–23]. Among them, the use of EPCs (CD34 + CD133+) from G-CSF-mobilized blood administrated intramuscularly in a small group of patients with no-option CLI resulted in a safe and feasible therapy for these patients, with pain reduction and increase of vascular perfusion [24, 25]. Overall, clinical trials of cell therapy for PAOD/CLI have shown promising results, but they are still in early-phase stages [21]. On the other hand, the high variability seen between the clinical results [21, 23], as well as the fact that EPC quantity and function seem to be impaired under pathological circumstances [26] makes compulsory the need to better understand how these cells work and participate in restoration of ischemic tissues in order to implement their clinical use. In this regard, in vitro and in vivo assays have shown that EPCs (both CACs and ECFCs) are susceptible to inflammatory and pro-atherogenic factors such as ox-LDL, GM-CSF, SDF-1, or IL-8, alone or combined, promoting an increased neovascularization of ischemic tissues with pre-stimulated cells [12, 27]. Very recently, we have shown that the incubation ex vivo of CACs with atherosclerotic factors promoted an increased mobilization of these cells, as well as an increased expression of vasculogenic-related markers [12].

In the current approach, we focused on the analysis of CACs, in order to confirm their involvement in tissue revascularization. Thus, an in vivo assay was performed, with CACs being administered to CLI mice, in order to identify the molecular changes detected in the ischemic tissues right after the injury and, moreover, in response to the treatment with CACs. By using a quantitative proteomic assay, we show protein changes promoted in the first days after cell administration within the damaged tissues. Furthermore, the proteins identified correlated with cell migration, angiogenesis, and other processes related to ischemia and vascular repair.

## Methods

### Cell isolation and culture

Circulating pro-angiogenic cells, hereafter referred to as CACs, were isolated from the peripheral blood of healthy donors, as previously described [12]. Briefly, fresh peripheral blood mononuclear cells (PBMCs) were isolated and plated in fibronectin-coated plates (10 μg/ml) and incubated in 2% FBS/EBM-2 media containing SingleQuots growth factors (Lonza). Non-adherent cells were discarded after 4 days, and attached cells were allowed to grow until day 7 in fresh media, when CACs were used for experiments.

### Cell characterization

Cell identity was confirmed by flow cytometry analysis, using specific antibodies against CD31, CD34, CD45, CD90, CD73, CD105, CD309, CD133-PE, CD146, and CD14 molecular markers, as described [17]. An isotype IgG1 antibody was used for negative control (Becton-Dickinson 345816). The full list of antibodies is shown in supplementary Table S5.

Additionally, uptake of acetylated LDL (ac-LDL), a function associated with endothelial cells, was assessed as previously described [12]. On day 7, cultured cells were incubated with 4 μg/mL DiI-Ac-LDL for 4 h. Cells were then fixed with 4% paraformaldehyde (PFA) and incubated with 10 μg/mL FITC-labeled *Ulex europaeus* agglutinin-I (UEA-1, lectin) for 1 h. DiI-Ac-LDL/lectin double-positive cells were identified as CACs.

### Animals

Balb-C Nude (CAnN.Cg-Foxn1nu/Crl) mice (n:28), age 9 weeks, were obtained from Charles River Laboratories. Mice were allocated in special rooms in specific cages, with technical staff, constantly supervising filters and air recirculation. Animals were fed sterile standard chow diet ad libitum and had free access to sterile water. Additionally, animals were constantly monitored to carry out euthanasia in case of excessive suffering or the presence of symptoms (such as infection) which would likely affect the experiment results. No animal was sacrificed prematurely during the experiment although one mouse from group SE4 died and was not included in the final analysis.

### CLI model and cell administration

Mice were randomly allocated between the groups. Mice were anesthetized with ketamine (100 mg/kg) and xylazine (10 mg/kg) administered intraperitoneally before surgery. Ischemia was induced in the left limb by double ligation of the femoral artery, occluding the distal and proximal ends of the femoral artery with double knots (non-absorbable 6/0) of suture, as described [28]. Additionally, mice received ketoprofen (2 mg/kg) intraperitoneally during 3 days after surgery.

Mice received 3–4 intramuscular injections in different sites of the left limb muscles, low back, low frontal, and middle muscles (supplementary figure S1), administrating either 5 × 10^5^ CACs in 50 μl physiological serum (treated group; SE, n:8) or 50 μl physiological serum without cells (ischemic group; SC, n:8), 24 h after surgery. Additionally, a healthy control (C, n:4) and a sham surgical control group (SH, n:4), undergoing femoral artery isolation, without ligation, were also used. Shams were used for proteomic assays while healthy controls were used in the analysis of vascular density changes.

Blood flow was measured at baseline for both paws, right after surgery and every day of the study, using a Laser Doppler system (Periflux System 5000; Perimed). Perfusion was expressed as a ratio of the left (ischemic) to right (non-ischemic) limb.

### Cell pre-labeling assay

In addition to the groups described in Section 2.4, another set of Balb-C Nude mice (n:8) were employed to confirm the presence of human CACs within the injured area, by using a pre-labeling approach. Thus, CACs (2 × 10^6^) were pre-labeled with Lectin-*Ulex europaeus* Agglutinin I (UEA1) (1:300), 1 h at 37 °C, and 5% CO_2_ and washed several times with PBS 1X before being administered (5 × 10^5^ cells/mouse) to mice that underwent femoral ligation 24 h earlier, as described above.

### Tissue extraction

**Fig. 1:**
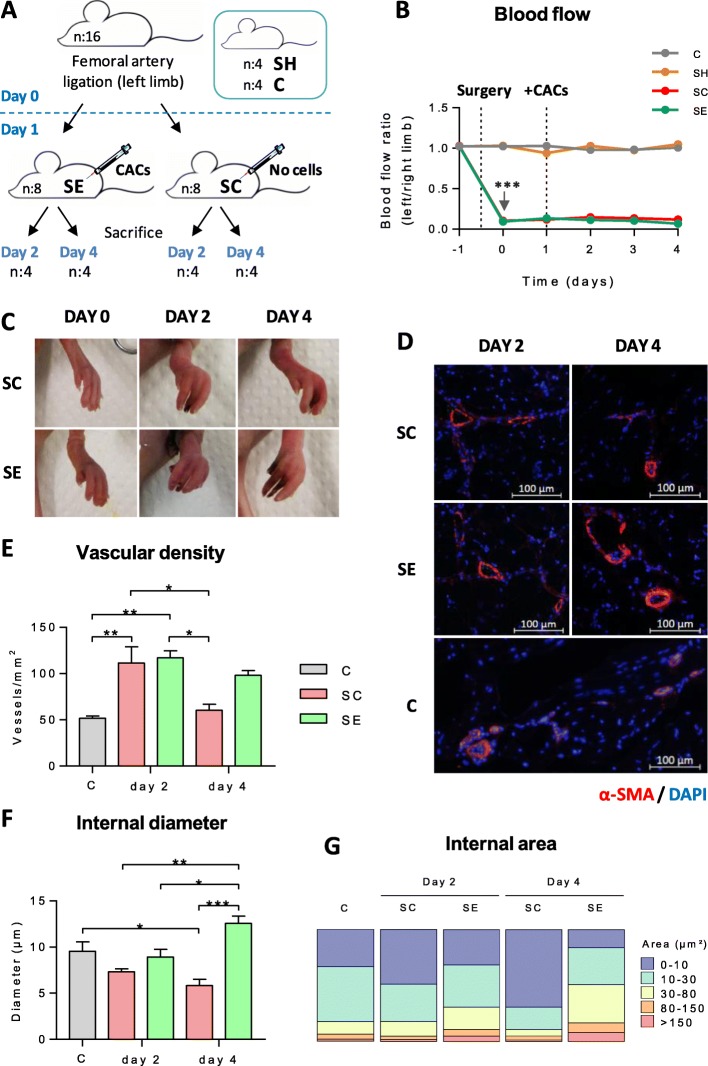
CLI progression, blood flow changes, and vasculogenesis in ischemic vs CAC-treated mice. **a** Schematic representation of experimental mice distribution. **b** Blow flow evolution per group (C, SH, SC, and SE) within time. Averaged ratios of the left (injured) vs right (non-injured) limbs are shown. **c** Representative images of ischemic symptoms (inflammation, necrotic fingers) in SE and SC mice. **d** IHC images to measure vascular density and diameter size, using anti-mouse smooth muscle α-actin (red) and DAPI (blue). **e** The number of vessels (vessels/mm^2^) and **f** diameter size (μm) were calculated in SE and SC groups vs C (healthy controls). **g** Vessel classification based on abundance percentage of different ranges of internal lumen areas (μm^2^). Groups analyzed: C: healthy control (n:4); SH: sham, surgery controls (n:4); SC: ischemic mice, no cell treatment (n:4); SE: ischemic mice, CAC treatment (day 2, n:4 and day 4, n:3). Data were presented as mean ± SEM. Significant differences were seen by two-way ANOVA (**b**) and one-way ANOVA (**e**, **f**) and Tukey post hoc in all cases. *p value < 0.05, **p value < 0.01, ***p value < 0.001

Mice from groups SE and SC were sacrificed (n:4 per group) on days 2 and 4 after surgery (SE2, SE4, SC2, and SC4), while the SH (sham) and C (healthy controls) were sacrificed on day 1 (Fig. 1a). Mice treated with pre-labeled CACs were also sacrificed on days 2 (n:4) and 4 (n:4). All mice were sacrificed in a CO_2_ chamber. For all of them, the muscles of the hind limb were harvested (supplementary figure S1): the low frontal muscles (tibialis) of the left limb were embedded in 10% formaldehyde for 15 days and 30% sucrose during 24 h, before OCT congelation for appropriate preservation prior immunohistochemistry (IHC). The low back (gastrocnemius and soleus) and middle muscles (bicep femoris, adductor, and semi-membranous) of the left limb were snap-frozen in liquid N_2_ and stored at − 80 °C for further Alu-based quantification and proteomic analysis.

### Immunohistochemistry analysis

IHC was performed with tissue sections from SE and SC groups, in order to detect human (h) cells and vessel formation after cell administration, as well as neutrophil mobilization toward the ischemic tissues. Thus, OCT embedded sections of the low frontal muscles (8 μm) coated in poly-lysine slides were pretreated with 1% SDS for 5 min for antigen retrieval and with 1% triton, 20 min, for permeabilization before blocking with 5% goat serum and 0.1% triton, 1 h. Then, tissue sections were incubated with primary antibodies overnight, labeling with UEA1 (1:300), anti-α-actin smooth muscle (1:500), anti-hCD31 (1:500), and anti-mouse Ly-6G (1:500) antibodies. After that, tissue slides were incubated with 0.3% Sudan Black B-70% ethanol for 20 min, to eliminate tissue auto-fluorescence. Further incubation with specific secondary antibodies for 1 h at room temperature (RT), in the dark, was carried out (see Table S5 for detailed information regarding antibodies). Finally, nuclear staining was done by incubation with DAPI (0.2 μg/ml).

In total, 5 tissue sections (8 μm), separated by 16 μm each, were used per mouse (from the low frontal muscles), analyzing the entire tissue areas of each section by fluorescence microscopy, for cellular and blood vessel detection. Images were acquired at × 20, using a MMI CellCut Plus (Olympus) and visualized with the Zen 2 (Zeiss) software. Results were expressed as the number of cells per square millimeter, the number of blood vessels per square millimeter, or blood vessel diameter (μm) and area (μm^2^). Additionally, the number of positive blood vessels with UEA1+ cells per total number of vessels was also quantified, as described [29]. Results were expressed as the mean ± SEM.

### Alu-based quantification

Human DNA (hDNA) was quantified by using the Alu-detection approach, as described [30]. Genomic DNA from the middle muscles was extracted with Quick-DNA™ Midiprep Plus Kit (ZymoResearch D4075) after Proteinase K (ZymoResearch D3001-2-B) treatment.

Alu sequences were amplified in 100 ng of genomic DNA by qPCR using TaqMan Universal Master Mix II (ThermoFisher 4440043), and 0.2 μM of primers and 0.25 μM of hydrolysis probes designed by [30]: forward primer 5′-GGTGAAACCCCGTCTCTACT-3′, reverse primer 5′-GGTTCAAGCGATTCTCCTGC-3′ and label probe 5′-(6-FAM)-CGCCCGGCTAATTTTTGTAT-(BHQ-1)-3′ (synthesized by Metabion). qPCR was performed using a CFX Connect Real-Time System (Biorad) with the following protocol: 1 cycle of 95 °C/10 min and 50 cycles of 95 °C/15s, 56 °C/30s and 72 °C/30s. CT values were analyzed with Bio-Rad CFX manager software (BioRad).

A standard curve was calculated with known amounts of hDNA (from 5 ng to 1 pg) mixed with murine DNA to determine the quantity of hDNA in experimental tissue samples, as described [30]. qPCR was performed in triplicates and Cq mean values were plotted to obtain the linear equation as well as the *R*^2^ values (supplementary figure S3).

The amount of hDNA detected in 100 ng of total genomic DNA extracted from the muscles was measured by qPCR, and further extrapolation of the total hDNA extracted was then calculated per milligram of the muscle. In the same way, the amount of DNA (ng) per cell was calculated, considering the relation of 5 pg of DNA per human cell [31]. Results were expressed as the mean ± SEM of hDNA per milligram of the muscle and human cells per milligram of the muscle detected.

### Proteomic analysis

The muscles from the left limbs were resuspended in lysis buffer (1% NP40, 50 mM HEPES pH 7, 150 mM NaCl, and 1 mM EDTA, supplemented with protease inhibitors) and homogenized by a mechanical procedure for protein extraction. Proteins (100 μg) were precipitated with 100% acetone overnight at − 20 °C. Pellets were resuspended in 6M, reduced (10 mM DTT), and alkylated (50 mM IAA) before in-solution digestion with Lys-C (Promega) (enzyme/substrate ratio 1:65) for 4 h at RT. Samples were diluted four times with 50 mM ammonium bicarbonate and trypsin digested (ThermoFisher) (enzyme/substrate ratio 1:50) at 37 °C overnight. Finally, the digestion was quenched with 0.1%TFA before peptide purification with C18 columns. Peptide mixtures were analyzed by nano-LC-MS/MS on an Orbitrap Q-Exactive HF-X (ThermoFisher) coupled to an EASY-LC 1000 system (Thermo Fischer Scientific). Peptides were loaded onto a 150 ID EASY-SPRAY column in A-buffer (0.1% formic acid). After washing, the peptides were eluted at a flow rate of 1.2 μl/min with an increasing gradient of B-buffer (95% acetonitrile in 0.1% formic acid). The gradient was stating at 2%B, going to 28%B in 80 min and to 40%B in 90 min. The Q-Exactive HFX was operated in data-dependent acquisition mode using the following settings: full-scan automatic gain control (AGC) target 3e6 at 120000 FWHM resolution, scan range 350–1400 m/z, orbitrap full-scan maximum injection time 100 ms, MS2 scan AGC target 1e5 at 15000 FWHM resolution, maximum injection 50 ms, normalized collision energy 30, dynamic exclusion time 20 s, isolation window 1.2 m/z, and 20 MS2 scans per MS full scan.

In order to identify proteins differently expressed between ischemic vs non-ischemic conditions and, moreover, in response to CAC treatment, all four groups (SE, SC, SH, and C) were compared, including time-related changes as well, n:4 per group (C, SH, SC2, SC4, SE2) and SE4 (n:3, one died before). Technical duplicates for each biological sample, SE and SC (n:4 per day) and SH and C (n:4), were run by LC-MS/MS.

Protein identification was performed using ProteomeDiscoverer 2.2 software (Thermo Fisher Scientific), searching against the Uniprot mouse database (taxonomy, mouse; 54,424 protein entries) using Mascot (version 2.3.0.2, Matrix Science Ltd). The following criteria were used: fixed modification: carbamidomethylation (C); dynamic modifications: oxidation (M), protein N-terminal acetylation, precursor mass tolerance 15 ppm; fragment mass tolerance 0.05 Da; enzyme: trypsin; and 2 missed cleavages were allowed. Data filtering was performed using a percolator, resulting in 1% false discovery rate (FDR). Additional filters were search engine rank 1 peptides and Mascot ion score > 20.

Additionally, an ion intensity-based label-free quantification was performed, comparing all groups between themselves and between times. Comparisons vs the sham group (SH) were made to discard changes due to surgery handling, while C was used as healthy, basal levels. Differentially expressed proteins were defined as follows: *p* value (*t* test) < 0.05 and fold-change rates > 1.5 for upregulated or < 0.6 for downregulated, for proteins identified in at least 2 replicates. Data processing and graphs were done with Perseus (hierarchical cluster), Proteome Discoverer (plots and PCA analysis), and Venny 2.1 (Venn’s diagram). Clustering was done with the following parameters: Euclidian distances for cluster conditions (upper tree) and Euclidean (normal) distance between proteins. The Ingenuity Pathway Analysis (IPA; Qiagen) platform was used for functional classification analysis of identified proteins.

### Validation of protein changes detected

In order to validate the MS data, the levels of Apo-E protein were measured by western blot (WB). Proteins from tissue lysates (15 μg) were resuspended in Laemmli sample buffer (50 mM Tris pH 6.8, 10% v/v glycerol, 2% w/v SDS, 0.1% w/v bromophenol blue) and separated on 4–15% polyacrylamide stain-free gels, transferred to a PVDF membrane (Biorad), blocked with 5% BSA for 1 h, RT, and immunoblotted with anti-Apo-E antibody (1:1000), 1 h at RT. After several washes with TBS-Tween-20 buffer (Tris-buffered saline: 10 mM Tris-Cl, pH 7.5, 150 mM NaCl 1X and 0.05% Tween 20) at RT, the membrane was incubated with secondary antibodies for 1 h at RT. Images were acquired using ChemiDoc Touch System (Biorad). An image from the Ponceau stained membrane was also taken as a loading control. Check supplementary Table S5 for detailed information regarding antibodies.

### Statistical analysis

Protein-related statistics were obtained with ProteomeDiscoverer 2.2 (Quantitative analysis) and IPA software, while functional-related statistical analysis was performed with GraphPad Prism 7 software. For functional assays, data were verified for normal distribution using Shapiro-Wilk normality test. The difference between the three groups (SE, SC, and either shams or healthy mice as controls) was assessed with either one-way ANOVA test and Tukey’s multiple comparisons test for post hoc analysis, or Kruskal-Wallis test and Dun’s test as post hoc analysis. Experiments with two different categorical independent variables (blood flow measurements within time) were analyzed with a two-way ANOVA test completed with Tukey’s multiple comparisons test for post hoc analyses. Data were presented as mean ± SEM and differences were considered statistically significant at *p* value < 0.05.

## Results

### Characterization of CACs

CACs were isolated and cultured as described [12]. By day 7, the ex vivo differentiated CACs were positive for the markers CD31, CD34, CD45, CD73, CD105, CD146, CD309, CD14, and CD133 and negative for CD90 (supplementary figure S2). Additionally, they were also double positive to FITC-UEA-1 and Ac-LDL, in agreement with previous reports, confirming the identity of these cells [32].

### Progression of ischemic symptoms

In response to femoral ligation, a significant reduction of blood flow (> 90%) was seen in all SE (ischemic, CAC-treated mice) and SC (ischemic, no treatment) mice right after surgery (*p* value < 0.001, Fig. 1b), compared to basal ratios of shams (SH, without ligation) and healthy controls (C), and these levels remained until day 4, when animals were sacrificed. On day 2, ischemic mice showed already symptoms of inflammation and ischemia (reddish area and black nails, reduced motility) and progressed to black necrotic fingers in some cases (Fig. 1c). In this regard, no significant differences were seen between SE and SC untreated mice, indicating that the expected effect of CACs was not detected in these initial phase, at least externally.

### CACs promote collateral vessel formation and arteriogenesis

In response to surgery, a significant increase in the number of vessels was seen on day 2 in SC and SE mice (*p* value < 0.05), with a similar increase in both groups, compared to the healthy group (C) (Fig. 1e). By day 4, however, vascular density levels remained higher only in the SE-treated mice, although not significantly compared to controls, while decreased significantly in SC mice compared to the values seen for SC and also SE on day 2 (*p* value < 0.05).

In terms of diameter size (Fig. 1d, f), no changes were seen on average on day 2 in the vessels of SC or SE groups vs controls, although diameters were slightly smaller in SC. On the other hand, the internal lumens on day 4 were wider in CAC-treated mice compared to day 2 (SE4 vs SE2, *p* value < 0.05) and, moreover, compared to SC non-treated mice (SC4 vs SE4, *p* value < 0.001, and SC2 vs SE4 *p* value< 0.01), while the diameters for the SC group were smaller than day 2 (not significantly) and smaller than controls (SC4 vs C, *p* value < 0.05).

Furthermore, classification based on the internal lumen size (Fig. 1g) indicated that SC mice had higher percentages of narrower vessels (< 10 μm) than controls and SE mice, with a decrease of the lumen area within time. On the other hand, SE mice showed lower percentages of small vessels (< 10 μm) while more vessels with bigger diameters (> 30 μm) compared to C and SC groups. Additionally, the number of wider vessels increased from day 2 to day 4 after cell administration.

### CACs migrate to the vessels of the ischemic area after intramuscular administration

**Fig. 2:**
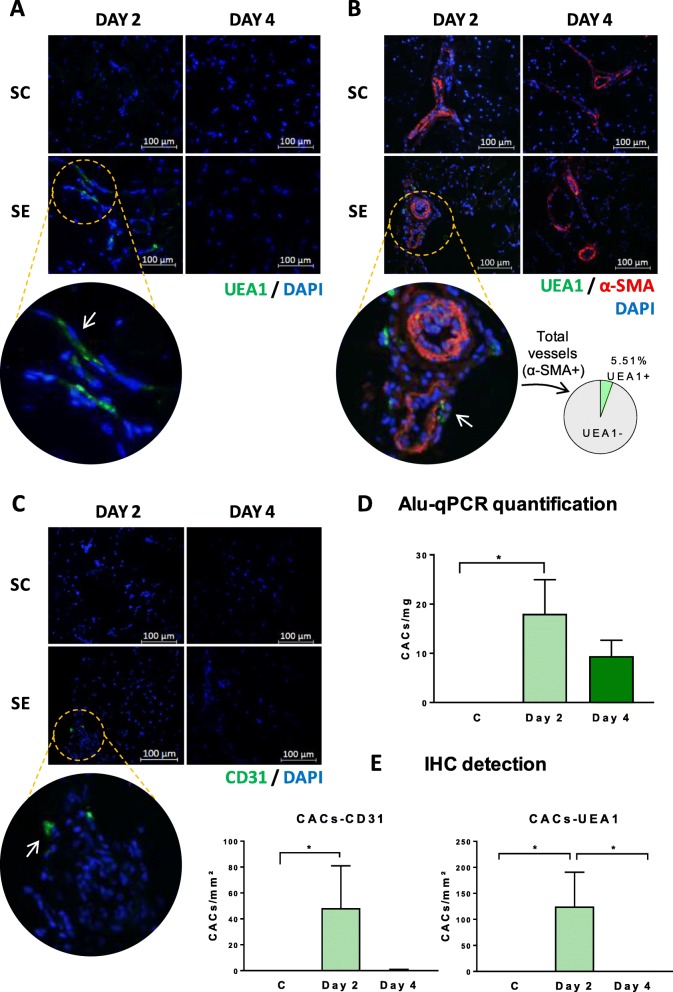
Detection of human cells in CAC-treated mice by IHC and qPCR. Human cells were analyzed by IHC in the frontal muscles of SE left limbs on days 2 and 4: **a** tissues infused with UEA1 pre-labeled cells, or tissues incubated with **b** UEA1 and anti-α-SMA antibody and **c** anti-hCD31, to determine the location of CACs in the tissue. **d** Graphical representation of the number of human cells (number of cells/μm^2^) detected after IHC labeling with anti-hCD31 and UEA1. **e** Quantification of human cells (number of cells/milligram of the tissue) by qPCR using human-specific Alu sequences, with the SH group as the negative control. Data represented as mean ± SEM, n:4 per day/group. Significant differences were seen by Kruskal-Wallis test and Dun’s test for multiple comparisons. *p value < 0.05

UEA1+ pre-labeled cells (directly administered, Fig. 2a) but also UEA1 and hCD31+ cells (after tissue post-staining, Fig. 2b, c) were found within the ischemic tissues of SE-treated mice on day 2, most of them in the vicinity of the blood vessels, demonstrating that these cells migrate to the vasculature even after intramuscular injection. In fact, from the total of the blood vessels detected on day 2, 5.51% of them had incorporated UEA1+ CACs. We did not detect human ECs by IHC on day 4.

The incorporation of human cells was also corroborated by in situ hybridization of human-specific Alu sequences (Fig. 2e), with hDNA detected on days 2 and 4. Nevertheless, Alu-quantification showed lower amounts of human material within the ischemic tissues on day 4 than on day 2, suggesting that the presence of infused cells decreased within time.

### Proteomic changes in response to ischemia and cell administration

**Fig. 3:**
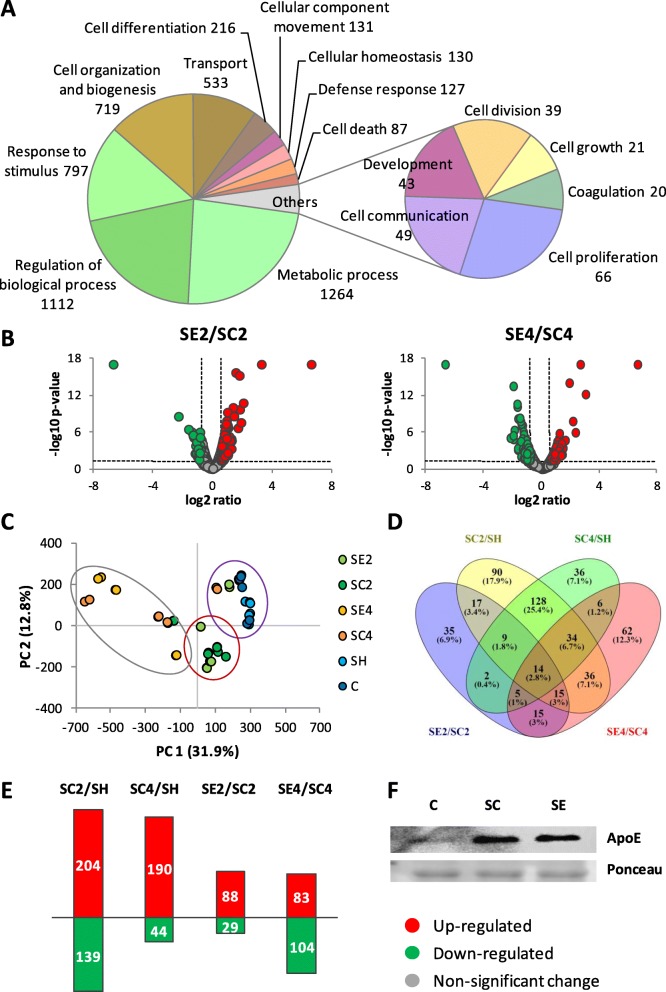
Proteomic changes in response to ischemia and CAC treatment. **a** Functional classification of identified proteins based on Gene Ontology Database (via ProteomeDiscoverer). **b** Volcano plots obtained after label free quantification, comparing protein levels in SE and SC, on days 2 and 4. Cut-off limits: *p* value < 0.05 and ratios for upregulated (> 1.5) or downregulated (< 0.6) proteins. Color legends: upregulated () or downregulated () proteins, no significant changes (). **c** Principal component analysis (PCA) graph distributed groups (SE, SC, SH, and C) mainly between controls and ischemic mice, but also per time (controls, day 2 and day 4). **d** Venn’s diagram showing the overlapping of proteins differently expressed between SC and SH and also between SE/SC on both days analyzed. **e** Number of proteins upregulated (red) and downregulated (green) by ischemia (SC/SH) and in response to CAC treatment (SE/SC) per day. **f** Validation by WB of proteomic results. As an example, the results seen for ApoE are shown. Apo-E was upregulated in SC and SE mice compared to controls

Derived from the proteomic analysis, 1938 proteins were identified, which were related to several biological processes according to Gene Ontology database (Fig. 3a), including among others, proteins related to cell organization and biogenesis (719), cell differentiation (216) and proliferation (66), cellular component movement (131), cell communication (49) and homeostasis (130), defense response (127), or cell death (87).

The application of a label-free quantitative approach allowed the identification of protein changes in response to ischemia and/or CAC treatment (Fig. 3b). Thus, differential protein expression was seen not only between the ischemic non-treated mice (SC) and the sham, non-ischemic mice on day 2 (343 proteins altered in SC2 vs SH) and day 4 (234 proteins in SC4 vs SH), but also between CAC-treated and SC non-treated mice, on day 2 (117 proteins altered in SE vs SC) and day 4 (187 proteins in SE vs SC). Additionally, a PCA classification based on differential protein expression clearly discriminated between controls and changes detected on days 2 and 4, suggesting that protein changes were also time-dependent (Fig. 3c). Supplementary Tables ST1 and ST2 contain complete information about these quantification data (abundance ratio and *p* value). As an example, to validate proteomic data, upregulation seen for Apo-E in both SC and SE mice compared to SH controls was confirmed by WB (Fig. 3f).

**Fig. 4:**
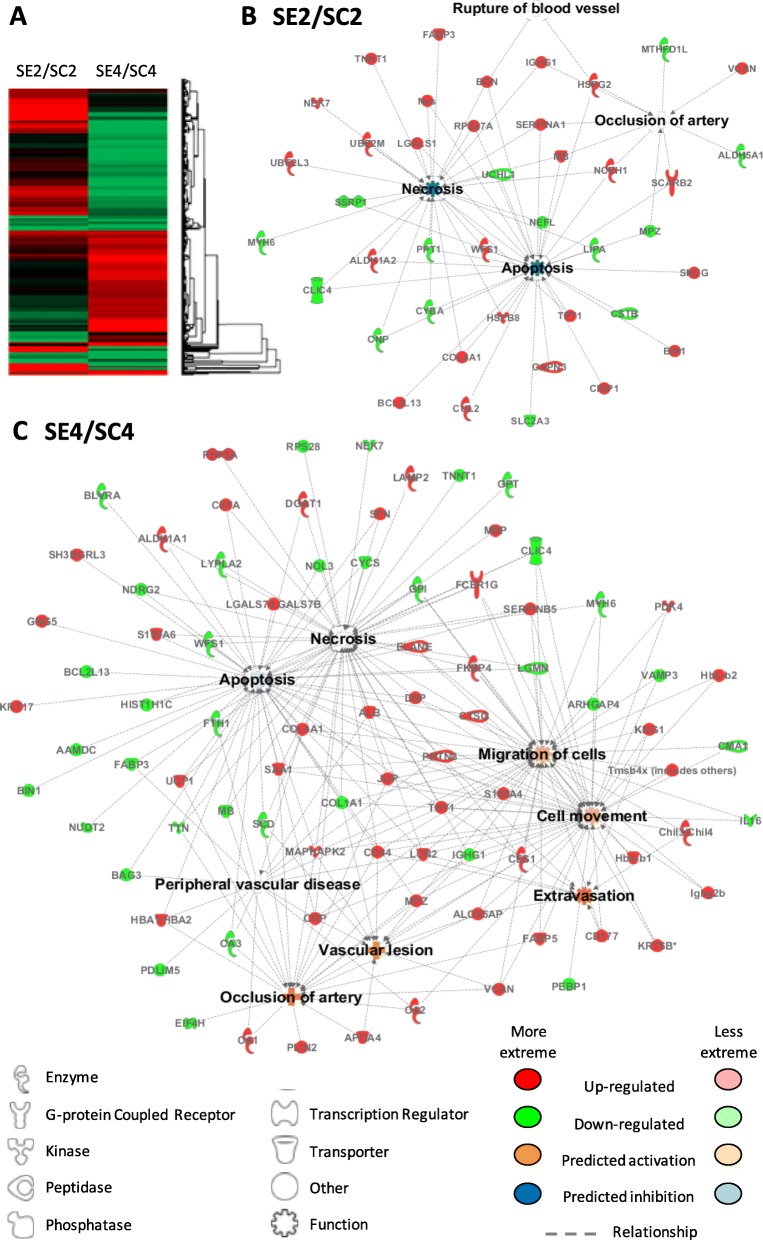
IPA functional networks for proteins differentially expressed in response to CAC treatment. **a** Hierarchical clustering of proteins differentially expressed in CAC-treated (SE) vs ischemic non-treated (SC) mice. Major functions described for protein changes (*p* < 0.05) seen in CAC-treated mice (SE) vs ischemic, non-treated mice (SC): **b** on day 2 and **c** day 4. Pathways were generated based on the information stored in the IPA Software Knowledge base. Color legends: upregulated () or downregulated () proteins, no significant expression changes (●), predicted activation (more  or less  confidence), and predicted inhibition (more  or less  confidence)

Interestingly, protein changes seen not only between ischemic (SC) vs non-ischemic (SH) mice, but also between SE ischemic-treated mice (SE vs SC) were associated according to IPA databases, with atherosclerosis, vascular lesion, artery occlusion, and PAOD, and also with angiogenesis, vasculogenesis and vasculature development, EC development, apoptosis, necrosis, cell movement, and cell migration (Fig. 4). Tables 1 and 2 include the functional classification made for protein changes on days 2 and 4 between SE and SC groups. Additionally, supplementary tables ST3 and ST4 include information regarding SC vs SH comparisons.

**Table 1:**
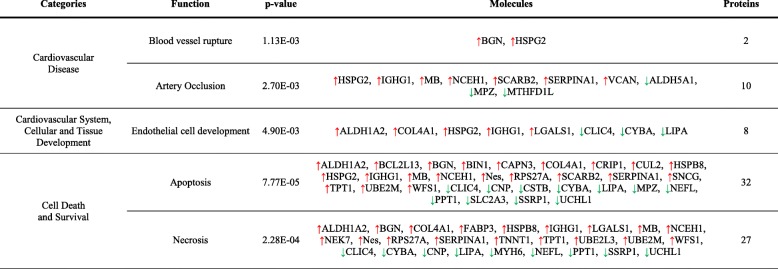
Functional classification of differentially expressed proteins in CAC-treated mice (SE) vs ischemic non-treated mice (SC) on day 2 after femoral ligation. Protein classification was made with the IPA software based on biomedical literature and integrated databases. The table shows the most probable pathways or functions in which the proteins of interest are involved including the main categories, the related functions or diseases, *p* value, molecules (protein names), and number of proteins per category. Legend:  upregulated,  downregulated

**Table 2:**
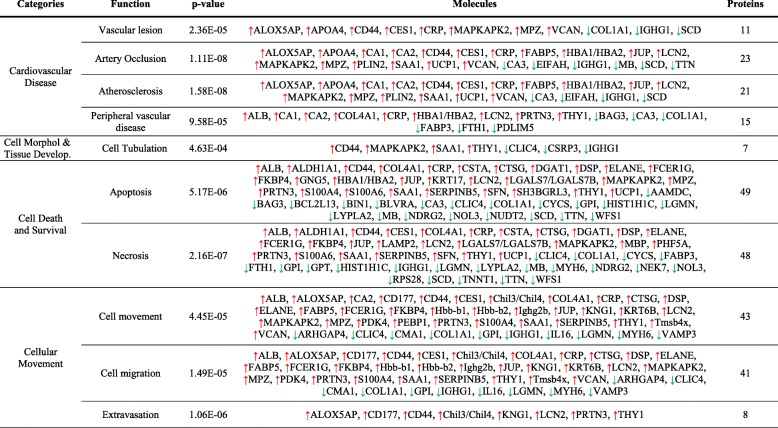
Functional classification of differentially expressed proteins in CAC-treated mice (SE) vs ischemic non-treated mice (SC) on day 4 after femoral ligation. Protein classification was made with the IPA software based on biomedical literature and integrated databases. The table shows the most probable pathways or functions in which the proteins of interest are involved including the main categories, the related functions or diseases, *p* value, molecules (protein names), and number of proteins per category. Legend:  upregulated,  downregulated

**Fig. 5:**
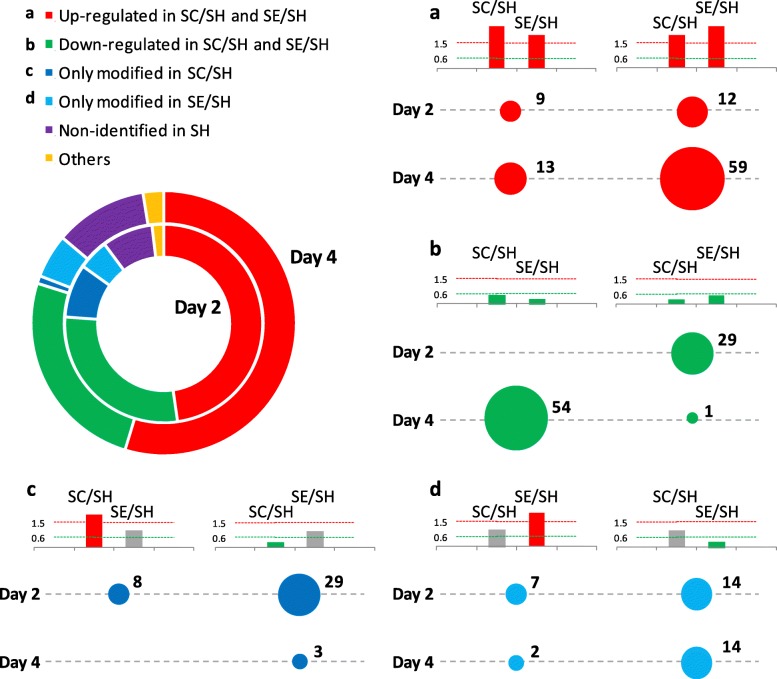
Differential protein expression patterns in CAC-treated mice and non-treated mice compared to shams. The circular chart represents the percentages of proteins altered in both ischemic groups (SE and SC) compared to shams (SH), on days 2 and 4: upregulated (red ) or downregulated proteins (green ) in SE and SC mice; proteins only modified in SC (dark blue ) or in SE mice (light blue ); proteins identified in ischemic mice (SC, SE) but not in SH (purple ); others changes (yellow ). Additionally, the number of proteins differently expressed, with different patterns within the groups described in the main chart is shown: upregulated (**a**) or downregulated (**b**) proteins in SE and SC vs SH, proteins only modified in SC (**c**) or in CAC-treated mice (**d**)

Detailed analysis indicated that most proteins were upregulated (48% on day 2 and 55% on day 4) or downregulated (28% and 25%) in both ischemic groups, SE and SC, compared to SH (Fig. 5 and Table 3). Among them, several proteins showed higher over-expression in response to CACs than SC (12 proteins in SE2/SC2, 59 SE4/SC4), while others were downregulated in lower levels after cell treatment (54 proteins, day 4). Additionally, some proteins were altered only in the ischemic, non-treated mice (27 proteins, SC2/SH and 3 proteins SC4/SH), while other proteins were only altered after cell treatment (21 proteins SE2/SH and 17 proteins SE4/SH). Finally, some proteins were identified only in the SE and SC but not in the SH group, probably as a result of the ligation procedure (Table 3).

**Table 3:**
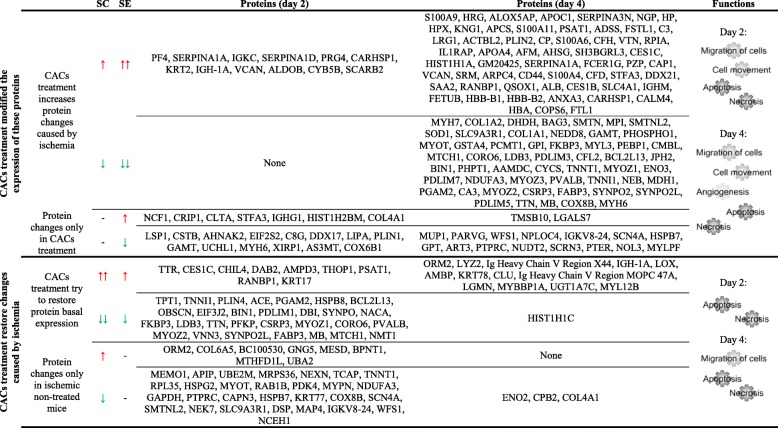
Classification of differentially expressed proteins in CAC-treated mice (SE) and non-treated mice (SC) vs shams (SH) on days 2 and 4 after femoral ligation. Protein classification was made based on the effect of CAC treatment over protein expression, compared to ischemic as well as basal levels (promoting higher changes or restoring proteins to basal levels). The table shows protein variations in SE and SC vs SH detected on day 2 and 4, the names of the proteins altered in each case as well as the most probably functions in which the proteins are involved, according to IPA-integrated databases. Legend:  upregulated,  downregulated

Overall, according to IPA analysis, protein changes in response to CACs affected cell death, with reduced apoptosis and necrosis from day 2 onwards, at the same time, that promoted cell movement and extravasation mainly from day 4 (Fig. 4). Such increase in cell movement was mainly related to immune cells: myeloid (granulocytes and monocytes) and lymphoid cells.

**Fig. 6:**
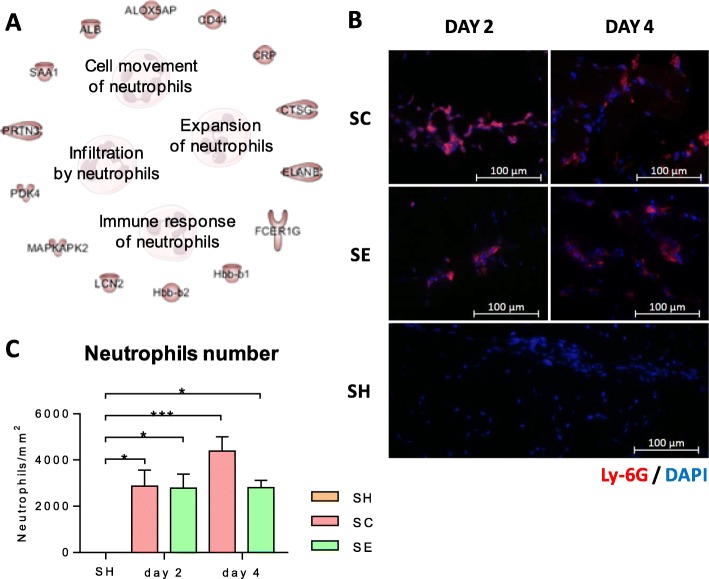
CAC administration modulates the recruitment of neutrophils to the ischemic tissues. **a** Functional analysis of the proteomic changes detected in response to CACs (SE vs SC, *p* < 0.05), indicated that these changes were involved in neutrophil recruitment (migration, adhesion, and transmigration) and neutrophil participation of the immune response. Pathways were obtained with the IPA software. **b** Detection of mouse neutrophils by IHC in the left hind limb frontal muscle labeling with Ly-6G antibody on days 2 and 4 in treated, non-treated, and sham mice. **c** Determination of neutrophils number (neutrophils/mm^2^) in treated, non-treated, and sham mice. Data were presented as mean ± SEM. Significant differences were found between SH (with no neutrophils) and the other groups: *p value < 0.05, ***p value < 0.001, by one-way ANOVA and Tukey post hoc test

In this regard, several proteins related to neutrophil migration, adhesion, and extravasation were differentially expressed in both SC and SE mice (Fig. 6a), suggesting an intense mobilization of these cells into the injured tissues. Indeed, IHC results confirmed the presence of a high number of neutrophils in SC and SE in response to ischemia (neutrophils were barely detected in shams, *p* values< 0.05). Interestingly, on day 4, the number of neutrophils increased even more in the SC mice, while remained at similar levels in the SE mice, compared to day 2 (Fig. 6c).

## Discussion

Experimental and clinical studies have shown that transplantation of BM-MNC- or PB-MNC-derived EPCs contribute to revascularization after ischemic events [26, 33, 34] improving angiogenesis and vasculogenesis within the ischemic tissues [35, 36], although the exact mechanisms remain still unclear. Growing evidence demonstrates that the so-called EPCs can be divided into two sub-types, early EPCs (also known as CACs or MACs) and ECFCs, and they both play a role in vascular homeostasis and endothelial repair [15, 17]. In this regard, validation of the regenerative potential of CACs and ECFCs is a pre-requisite step to their application, alone or combined, in clinical practice [15].

Herein, we focused on the role of CACs in CLI, taking into account that these cells seem to promote a significant recovery of blood flow after 7 days of being administered in CLI animal models [37–41], although they are barely detected after the second week. Thus, we postulated that administration of CACs must exert an effect at the molecular level right on the first days after their mobilization into the injured area, and such effect has to promote, in the last term, the increased revascularization observed, while the cells themselves are most probably replaced by the host ones or simply do not proliferate and die after having exerted their angiogenic role.

In our study, a significant blood flow reduction was seen after artery ligation (> 90%), and these levels remained until day 4, when mice were sacrificed. Additionally, ischemic symptoms such as limb inflammation and necrotic nails were also seen, corroborating the ischemic process induced. As a result, an increase in vascular density was detected on day 2 in the ischemic mice (SE and SC), reflecting an expected sprouting of angiogenic ECs in response to injury as a result of reduced oxygen delivery into the damaged tissues [42]. On day 4, however, only CAC-treated mice maintained the vascularity enhanced after ischemia, showing as well a higher percentage of the vessels with wider diameters than SC or the healthy controls. All these data suggest that, despite not having seen an initial recovery of blood flow, cells start to exert their effect from the first moment they are into the ischemic tissues, promoting an initial revascularization by increasing the number of the vessels and, moreover, enhancing the maturation of the newly forming vasculature [43]. Moreover, the presence of human CACs was confirmed by Alu-quantification of hDNA and IHC assays, mainly on day 2, and in fewer amounts on day 4, many of them detected in the vessels even after intramuscular administration. Taking into account that CACs are not highly proliferative [21], such decrease should not be surprising, although future research should confirm the in vivo fate of these cells. Nevertheless, the decrease in the number of administered cells within time corroborated the assumption that, although CACs mobilize to the injured area, they do not participate themselves in the formation of the new vessels [44], but they most probably contribute to revascularization by triggering specific molecular changes in the ischemic tissues, as stated before [45], by inducing further mobilization of autologous cells, promoting angiogenesis and/or arteriogenesis itself. In this regard, the paracrine role assigned to CACs, known to secrete angiogenic factors such as VEGF, HGF, G-CSF, or GM-CSF [32], could help to explain those changes.

Indeed, the further proteomic analysis identified not only time-dependent protein variations but also changes in response to ischemia (SC) and, moreover, after CAC administration (SE). Thus, in response to CACs, a tendency to restore proteins downregulated by ischemia to basal levels was seen in the CLI-treated mice on day 2 (SC2 vs SE2) for proteins such as Desmoplakin (DSP) or Microtubule-associated protein 4 (MAP 4). DSP participates in microvascular tube formation, taking part in de novo capillary formation and branching during cardiovascular development [46] while MAP 4 promotes migration and proliferation of ECs under hypoxia [47]. Additionally, changes detected on day 4 in SE mice represented an increased response against ischemia, for example, with proteins related to oxidative stress and nitric oxide (NO) bioavailability, a molecule with vasculo-protective properties, and an important role in angiogenic response after ischemia [33]. Among them, heme-related proteins were found either upregulated (hemoglobin, HB) or downregulated (myoglobin, MB) in ischemic tissues, and even more after CAC treatment (compared to controls). In the case of HB, two different subunits (HB-alpha and HB-beta) were identified upregulated in both SC and SE compared to shams, and moreover, these subunits were present in higher levels in the SE-treated mice on day 4. Both subunits are required for the proper functioning of HB as oxygen transport. Thus, an increase of HB levels could help to increase oxygen transport after the hypoxic situation induced by ischemia. Additionally, HB increase could promote vascular tone through participating in the NO transfer between ECs and vascular smooth muscle cells [48]. On the other hand, decreased levels of MB, a muscle-specific protein of oxidative metabolism, could also induce NO bioavailability [49, 50]. Hemopexin (HPX), another protein related to oxidative stress, was also upregulated after ischemia and CAC treatment. HPX has a strong affinity for free heme in plasma and can remove excessive free heme that excess might be strongly cytotoxic for vascular ECs [51].

Overall, according to the IPA software (which allows determining the most likely pathways or functions in which proteins of interest are involved, based on biomedical literature and integrated databases), the femoral ligation performed and, moreover, CAC treatment, induced a cascade of proteomic changes related not only to vasculogenesis, angiogenesis, cell migration, and tubule formation, but also to cell death, with clear correlation with atherosclerosis as well as PAOD. Thus, a significant increase of proteins related to cell movement was seen on day 4, especially for immune cells (myeloid cells, lymphoid cells, and phagocytes) in SC and, moreover, in SE mice. More intensively, CAC treatment seemed to affect neutrophil mobilization, with several related proteins upregulated on day 4, such as CD177, lipocalin-2 (LCN2) or CD44. In response to injury, neutrophils are usually the first cells infiltrating the damaged area, mediating inflammation by releasing diverse metabolites and pro-inflammatory cytokines against ischemia [52]. Thus, not surprisingly, a high mobilization of neutrophils was detected already on day 2 in both ischemic groups (SC and SE) but not in shams. Interestingly, however, the number of neutrophils continued increasing on day 4 only in the ischemic, non-treated mice (SC) but not in the CAC-treated ones. Although neutrophils are vital to fight inflammatory agents, excessive accumulation can also cause further injury by releasing high concentrations of cytolytic and cytotoxic molecules into the damaged muscles and surrounding areas [53, 54]. Diverse studies have indicated that the modulation of neutrophil functions constitute a complex network based on the balance between stimulatory and inhibitory pathways regulated by certain mediators (including cytokines, “classical” neuroendocrine hormones, and bioactive lipids) [55]. According to our results, the administration of CACs promoted changes at a protein level that, on average, avoided an excessive neutrophil infiltration into the ischemic tissues. Thus, the upregulation of CD177 or LCN2 might induce neutrophil migration in CAC-treated mice. CD177, a neutrophil-specific antigen, promotes neutrophil migration in endothelium by interacting with platelet endothelial cell adhesion molecule-1 (PECAM-1) [56, 57]. In the same way, LCN2, also known as neutrophil gelatinase-associated lipocalin (NGAL), could significantly contribute to neutrophil’s migration, since it constitutes an important paracrine chemoattractant and a key factor for neutrophil function in inflammation [58]. On the other hand, upregulation of proteins like Alpha1-Antitrypsin, also referred to as α_1_-proteinase inhibitor or SerpinA1, might help to explain the reduction of neutrophil infiltration seen on day 4 in response to CACs. SerpinA1 is an acute-phase protein, the most abundant serine proteinase inhibitor in human plasma, and, moreover, a potent regulator of neutrophil activation via both protease inhibitory and non-inhibitory functions [59]. Indeed, SerpinA1 can influence neutrophil chemotaxis preventing neutrophil activation independently to the proteinase role [60]. More specifically, two isoforms of SerpinA1, SerpinA1D and SerpinA1A, appeared upregulated, in higher levels, in SE2 and SE4, respectively, compared to SH and, moreover, compared to SC mice (Table 3). Further research will help to elucidate the exact role of these protein isoforms as well as the mechanisms through which CACs seem to modulate neutrophil infiltration in order to avoid, perhaps an excessive and unwanted inflammatory response.

On the other hand, neutrophils not only participate in inflammation but they can also promote vascularization by inducing angiogenesis via a pro-angiogenic phenotype [61, 62]. In this regard, proteins such as LCN2 have been found to induce angiogenesis in several breast cancers [63] and upregulation of LCN2 has been shown to promote EC tube formation and migration via iron and ROS-related pathways in rat’s brain [64]. Moreover, LCN2 is increased in atherosclerosis and upregulated by hypoxia and MI [65], being actually considered a potential biomarker of early ischemic acute kidney injury [66]. Additionally, the over-expression of the glycoprotein CD44 in the ischemic tissues and, mainly after CAC administration, could also represent a mechanism to promote angiogenesis as well as protecting the tissue from ischemia itself. CD44 is a cell surface protein expressed on adult ECs but also in CACs and other bone marrow-derived cells [67], where it works as a receptor for hyaluronan, a molecule related with blood vessel formation. Indeed, CD44/hyaluronan interaction seems to be important to activate relevant angiogenic signals [67, 68] including, among others, the mobilization and further adhesion of neutrophils to ECs, promoting the recruitment of these cells into the damaged areas after the ischemic process. CD44 could also participate in the organization and stability of endothelial tubular networks and EC proliferation [67, 68]. CD44 can also bind to the proteoglycan Versican (VCAN), which was upregulated in SC but even more in SE mice, and this over-expression increased on day 4. VCAN/CD44 interaction appears to be involved in extracellular matrix remodeling, cell adhesion, proliferation, migration, and survival, as well as angiogenesis and maintenance of tissues during vascular diseases [69, 70]. In addition, upregulation of Leucine-rich alpha-2-glycoprotein 1 (LRG1) could correlate with the capability of activated neutrophils to release LRG1 from the granule compartment and thus modulate the microenvironment in a paracrine fashion [71]. Alternatively, LRG1 can also promote cell proliferation, migration, and angiogenesis, by interacting with the TGF-β accessory receptor endoglin, thus modulating endothelial TGF-β signaling pathways and collaborating in blood vessel formation [72, 73].

Apart from neutrophils, monocytes and macrophages are required for the immune response and also induce the growth and maturation of small collateral arteries into conduit vessels (arteriogenesis), essential for restoration of blood flow to ischemic organs [74]. In this regard, upregulation of proteins such as MAP kinase-activated protein kinase 2 (MAPKAPK2) *by CACs represents an attempt to promote arteriogenesis, since this protein plays a key role in this process by inducing,* during early phases of hind limb ischemia, the production of monocyte chemo-attractant *protein*-1 (MCP-1) by ECs, and thus, monocyte migrates to the damaged area [74]. MAPKAPK2 increase could therefore be involved in monocyte/macrophage mobilization and the increase of vessel diameters seen after CAC administration.

Several members of the S100 family (S100A4, S100A6, S100A9, and S100A11) were also found upregulated in ischemia and more intensively in response to cell treatment. This family of calcium-binding cytosolic proteins has a broad range of intracellular and extracellular functions participating in proliferation, differentiation, apoptosis, Ca^2+^ homeostasis, energy metabolism, inflammation, leukocyte adhesion and migration, and tissue repair [75, 76]. Among others, the release of S100A8/A9 has been suggested to facilitate monocyte and neutrophil transmigration. These proteins are also considered as damage-associated molecular pattern (DAMP) molecules, since they appear to be released by damaged or stressed cells becoming danger signals that derive in the activation of immune and ECs by binding to the pattern recognition receptors such as TLRs and RAGE [76]. At relatively high concentrations, the S100A8/A9 complex inhibits the growth of a variety of normal cell types (macrophages, bone marrow cells, lymphocytes, fibroblasts) [75]. As DAMP molecules, S100 proteins also participate as either pro- or anti-apoptotic proteins depending on the cell type as well as the pathological process they are involved in. For example, downregulation of S100A6 has been related with an increased cell cycle arrest in the G2/M phase of cell cycle and downregulation of genes with effects on cell cycle progression [75, 77].

Not only S100A6 but also other altered levels of pro- and anti-apoptotic proteins could be found in the first days in SC and SE mice, with downregulation (more significant in SE) of pro-apoptotic proteins like mitochondrial carrier homolog 1 (MTCH1), B cell lymphoma (BCL)-2 like protein 13 (BCL2L13) [78] or anti-apoptotic ones like BCL2-associated Athanogene 3 (BAG3), which also promotes autophagy by associating with BCL-2 or Beclin 1 [79]. Apoptosis is triggered in ischemic tissues in order to remove damaged or unhealthy cells, accompanied by the repair of lost tissues via the active proliferation of the intact cell components. Autophagy, on the other hand, is an adaptive response to environmental stresses including nutrient starvation due to tissue destruction or blood shortage, and it seems to be also required for the development of vascular ECs [80]. Downregulation of BAG3 could represent an increase in apoptosis but reduced autophagy [79, 81], mainly in the CAC-treated mice, although further studies should confirm such hypothesis. Overall, protein changes (upregulation and downregulation) taking place in ischemic non-treated mice indicated an upregulation of apoptosis and necrosis (according to the IPA functional analysis), while cell treatment seemed to modulate such phenomena. Similarly, transplantation of human peripheral blood-derived CD31^+^ cells significantly reduced endothelial cell apoptosis compared to control groups suggesting protective effects on existing vessels [82]. Angiogenic factors not only induce angiogenesis but also act as a survival factor and can potentially block endothelial cell apoptosis and protect microvascular endothelium from programmed cell death [83, 84]. The imbalance seen between apoptosis/necrosis and cell proliferation could help to explain why in the absence of such angiogenic promoters, ECs die without being able to proliferate [84] while CAC treatment induced in SE mice has a stable cell sprouting and/or neovascularization as well as maturation of an appropriate vasculature network, probably modulating apoptosis to allow revascularization and blood flow recovery.

Overall, we have shown here, for the first time to our knowledge, a complete map of protein/molecular changes taking place right after inducing ischemia in a CLI model and, moreover, in response to CAC treatment. A functional classification has allowed to provide an overview of the proteins identified herein. Many of them have been previously correlated with response to ischemia (MAP 4, HPX, S100A proteins) as well as with angiogenic- and revascularization-related processes (LCN2, LRG1, CD44, MAPKAPK2). Other proteins will require further evaluation to confirm whether they promote indeed the effects described. Our findings bring the opportunity for future research to explore the precise role of the differential proteins identified in response to CAC administration.

## Conclusions

Our data confirmed that CACs migrate to the vasculature of the injured area, contributing to the formation of the new vessels, although they are most probably replaced by autologous ECs or simply disappear; due to the short time, they can be traced within the ischemic tissues, supporting the paracrine role assigned to these cells. Furthermore, the protein changes identified indicate that, from the first moment, CACs seem to trigger a set of mechanisms ranging from the recruitment of immune and other cells (participating in the inflammatory response but also contributing to vascular remodeling), vessel formation, and arteriogenesis (as it can be seen in the first days already), as well as cell apoptosis, probably to modulate an appropriate development of the vascular network.

## Supplementary information

**Additional file 1: Supplementary Figure S1.** CACs administration sites and muscle extraction. **Supplementary Figure S2.** Characterization of cultured CACs. **Supplementary Figure S3.** Standard curve for detecting human Alu sequence among the ischemic tissues. **Supplementary Figure S4.** Graphical correlations between differentially expressed proteins and functions due to ischemia, on day 2. **Supplementary Figure S5.** Graphical correlations between differentially expressed proteins and functions due to ischemia, on day 4. **Supplementary Table 1.** Quantitative comparison of proteins differentially expressed by ischemia on days 2 and 4 (SC vs SH). **Supplementary Table 2.** Quantitative comparison of proteins differentially expressed after CACs treatment on days 2 and 4 (SE vs SC). **Supplementary Table 3.** Functional classification of proteins differentially expressed by ischemic non-treated mice (SC) vs Shams (SH) on day 2 after femoral ligation. **Supplementary Table 4.** Functional classification of differentially expressed proteins in ischemic non-treated mice (SC) vs Shams (SH) on day 4 after femoral ligation. **Supplementary Table 5.** List of antibodies used in this study. **Supplementary table 6.** Secondary antibodies used in this study.

## Data Availability

MS data have been deposited into the ProteomeXchange Consortium via the PRIDE [85] partner repository with the dataset identifier (PXD017661).

## Abbreviations

BAG3: Anti-apoptotic ones like BCL2-associated athanogene 3
BCL21L3: B cell lymphoma (BCL)-2 like protein 13
BM: Bone marrow
CACs: Circulating angiogenic cells
CLI: Critical limb ischemia
CVD: Cardiovascular disease
DAMP: Damage-associated molecular pattern
DAPI: 4′,6-Diamidine-2′-phenylindole dihydrochloride
DSP: Desmoplakin
ECFCs: Endothelial colony-forming cells
ECs: Endothelial cells
EPCs: Endothelial progenitor cells
G-CSF: Granulocyte colony-stimulating factor
GM-CSF: Granulocyte-macrophage colony-stimulating factor
HB: Hemoglobin
HGF: Hepatocyte growth factor
HPX: Hemopexin
IAA: Iodoacetamide
IHC: Immunohistochemistry
IPA: Ingenuity pathway analysis
LCN2: Lipocalin-2
LRG1: Leucine-rich alpha-2-glycoprotein 1
MACs: Myeloid angiogenic cells
MAP 4: Microtubule-associated protein 4
MAPKAPK2: MAP kinase-activated protein kinase 2
MB: Myoglobin
MCP-1: Monocyte chemo-attractant protein-1
MI: Myocardial infarction
MS: Mass spectrometry
MTCH1: Mitochondrial carrier homolog 1
NGAL: Neutrophil gelatinase-associated lipocalin
NO: Nitric oxide
PAOD: Peripheral arterial obstructive disease
PB-MNC: Peripheral blood mononuclear cells
PECAM-1: Platelet endothelial cell adhesion molecule-1
UEA1: *Ulex Europaeus* Agglutinin 1
VCAN: Versican
VEGF: Vascular endothelial growth factor
WB: Western blot

## References

[1] Krishna SM, Moxon JV, Golledge J (2015). A review of the pathophysiology and potential biomarkers for peripheral artery disease. Int J Mol Sci.

[2] Fowkes FG, Rudan D, Rudan I, Aboyans V, Denenberg JO, McDermott MM (2013). Comparison of global estimates of prevalence and risk factors for peripheral artery disease in 2000 and 2010: a systematic review and analysis. Lancet.

[3] Conte MS, Pomposelli FB (2015). Society for Vascular Surgery Practice guidelines for atherosclerotic occlusive disease of the lower extremities management of asymptomatic disease and claudication. Introduction J Vasc Surg.

[4] Simpson EL, Kearns B, Stevenson MD, Cantrell AJ, Littlewood C, Michaels JA (2014). Enhancements to angioplasty for peripheral arterial occlusive disease: systematic review, cost-effectiveness assessment and expected value of information analysis. Health Technol Assess.

[5] Walter DH, Krankenberg H, Balzer JO, Kalka C, Baumgartner I, Schluter M (2011). Intraarterial administration of bone marrow mononuclear cells in patients with critical limb ischemia: a randomized-start, placebo-controlled pilot trial (PROVASA). Circ Cardiovasc Interv.

[6] Jude EB, Oyibo SO, Chalmers N, Boulton AJ (2001). Peripheral arterial disease in diabetic and nondiabetic patients: a comparison of severity and outcome. Diabetes Care.

[7] Dragneva G, Korpisalo P, Yla-Herttuala S (2013). Promoting blood vessel growth in ischemic diseases: challenges in translating preclinical potential into clinical success. Dis Model Mech.

[8] Norgren L, Hiatt WR, Dormandy JA, Nehler MR, Harris KA, Fowkes FG (2007). Inter-Society Consensus for the Management of Peripheral Arterial Disease (TASC II). J Vasc Surg.

[9] Qadura M, Terenzi DC, Verma S, Al-Omran M, Hess DA (2018). Concise review: cell therapy for critical limb ischemia: an integrated review of preclinical and clinical studies. Stem Cells.

[10] Anderson JL, Halperin JL, Albert NM, Bozkurt B, Brindis RG, Curtis LH (2013). Management of patients with peripheral artery disease (compilation of 2005 and 2011 ACCF/AHA guideline recommendations): a report of the American College of Cardiology Foundation/American Heart Association Task Force on Practice Guidelines. Circulation.

[11] Ko SH, Bandyk DF (2014). Therapeutic angiogenesis for critical limb ischemia. Semin Vasc Surg.

[12] Vega FM, Gautier V, Fernandez-Ponce CM, Extremera MJ, Altelaar AFM, Millan J (2017). The atheroma plaque secretome stimulates the mobilization of endothelial progenitor cells ex vivo. J Mol Cell Cardiol.

[13] Asahara T, Murohara T, Sullivan A, Silver M, van der Zee R, Li T (1997). Isolation of putative progenitor endothelial cells for angiogenesis. Science.

[14] Patel J, Donovan P, Khosrotehrani K (2016). Concise review: functional definition of endothelial progenitor cells: a molecular perspective. Stem Cells Transl Med.

[15] Edwards N, Langford-Smith AWW, Wilkinson FL, Alexander MY (2018). Endothelial progenitor cells: new targets for therapeutics for inflammatory conditions with high cardiovascular risk. Front Med (Lausanne).

[16] Chopra HH, Hung MK, Kwong DL, Zhang CF, Pow EHN (2018). Insights into endothelial progenitor cells: origin, classification, potentials, and prospects. Stem Cell Int.

[17] Medina RJ, Barber CL, Sabatier F, Dignat-George F, Melero-Martin JM, Khosrotehrani K (2017). Endothelial progenitors: a consensus statement on nomenclature. Stem Cells Transl Med.

[18] Prater DN, Case J, Ingram DA, Yoder MC (2007). Working hypothesis to redefine endothelial progenitor cells. Leukemia.

[19] Banno K, Yoder MC (2018). Tissue regeneration using endothelial colony-forming cells: promising cells for vascular repair. Pediatr Res.

[20] Sabatier F, Camoin-Jau L, Anfosso F, Sampol J, Dignat-George F (2009). Circulating endothelial cells, microparticles and progenitors: key players towards the definition of vascular competence. J Cell Mol Med.

[21] Fujita Y, Kawamoto A (2017). Stem cell-based peripheral vascular regeneration. Adv Drug Deliv Rev.

[22] Sprengers RW, Lips DJ, Moll FL, Verhaar MC (2008). Progenitor cell therapy in patients with critical limb ischemia without surgical options. Ann Surg.

[23] Rigato M, Monami M, Fadini GP (2017). Autologous cell therapy for peripheral arterial disease: systematic review and meta-analysis of randomized, nonrandomized, and noncontrolled studies. Circ Res.

[24] Lara-Hernandez R, Lozano-Vilardell P, Blanes P, Torreguitart-Mirada N, Galmes A, Besalduch J (2010). Safety and efficacy of therapeutic angiogenesis as a novel treatment in patients with critical limb ischemia. Ann Vasc Surg.

[25] Tanaka R, Masuda H, Kato S, Imagawa K, Kanabuchi K, Nakashioya C (2014). Autologous G-CSF-mobilized peripheral blood CD34+ cell therapy for diabetic patients with chronic nonhealing ulcer. Cell Transplant.

[26] Sukmawati D, Tanaka R (2015). Introduction to next generation of endothelial progenitor cell therapy: a promise in vascular medicine. Am J Transl Res.

[27] Du F, Zhou J, Gong R, Huang X, Pansuria M, Virtue A (2012). Endothelial progenitor cells in atherosclerosis. Front Biosci (Landmark Ed).

[28] Niiyama H, Huang NF, Rollins MD, Cooke JP. Murine model of hindlimb ischemia. J Vis Exp. 2009;(23):1035. 10.3791/1035 PMC2763292 19229179

[29] van Weel V, van Tongeren RB, van Hinsbergh VW, van Bockel JH, Quax PH (2008). Vascular growth in ischemic limbs: a review of mechanisms and possible therapeutic stimulation. Ann Vasc Surg.

[30] Funakoshi K, Bagheri M, Zhou M, Suzuki R, Abe H, Akashi H (2017). Highly sensitive and specific Alu-based quantification of human cells among rodent cells. Sci Rep.

[31] Lee RH, Hsu SC, Munoz J, Jung JS, Lee NR, Pochampally R (2006). A subset of human rapidly self-renewing marrow stromal cells preferentially engraft in mice. Blood.

[32] Rehman J, Li J, Orschell CM, March KL (2003). Peripheral blood “endothelial progenitor cells” are derived from monocyte/macrophages and secrete angiogenic growth factors. Circulation.

[33] Zampetaki A, Kirton JP, Xu Q (2008). Vascular repair by endothelial progenitor cells. Cardiovasc Res.

[34] Ouma GO, Zafrir B, Mohler ER, Flugelman MY (2013). Therapeutic angiogenesis in critical limb ischemia. Angiology.

[35] Taguchi A, Soma T, Tanaka H, Kanda T, Nishimura H, Yoshikawa H (2004). Administration of CD34+ cells after stroke enhances neurogenesis via angiogenesis in a mouse model. J Clin Invest.

[36] Chen H, Wang S, Zhang J, Ren X, Zhang R, Shi W (2014). A novel molecule Me6TREN promotes angiogenesis via enhancing endothelial progenitor cell mobilization and recruitment. Sci Rep.

[37] Vaughan EE, Liew A, Mashayekhi K, Dockery P, McDermott J, Kealy B (2012). Pretreatment of endothelial progenitor cells with osteopontin enhances cell therapy for peripheral vascular disease. Cell Transplant.

[38] Hsu SL, Yin TC, Shao PL, Chen KH, Wu RW, Chen CC (2019). Hyperbaric oxygen facilitates the effect of endothelial progenitor cell therapy on improving outcome of rat critical limb ischemia. Am J Transl Res.

[39] Shen WC, Liang CJ, Wu VC, Wang SH, Young GH, Lai IR (2013). Endothelial progenitor cells derived from Wharton’s jelly of the umbilical cord reduces ischemia-induced hind limb injury in diabetic mice by inducing HIF-1alpha/IL-8 expression. Stem Cells Dev.

[40] Kalka C, Masuda H, Takahashi T, Kalka-Moll WM, Silver M, Kearney M (2000). Transplantation of ex vivo expanded endothelial progenitor cells for therapeutic neovascularization. Proc Natl Acad Sci U S A.

[41] Yoon CH, Hur J, Park KW, Kim JH, Lee CS, Oh IY (2005). Synergistic neovascularization by mixed transplantation of early endothelial progenitor cells and late outgrowth endothelial cells: the role of angiogenic cytokines and matrix metalloproteinases. Circulation.

[42] Cooke JP, Losordo DW (2015). Modulating the vascular response to limb ischemia: angiogenic and cell therapies. Circ Res.

[43] Watson EC, Grant ZL, Coultas L (2017). Endothelial cell apoptosis in angiogenesis and vessel regression. Cell Mol Life Sci.

[44] Yang JX, Pan YY, Wang XX, Qiu YG, Mao W (2018). Endothelial progenitor cells in age-related vascular remodeling. Cell Transplant.

[45] Yang Z, von Ballmoos MW, Faessler D, Voelzmann J, Ortmann J, Diehm N (2010). Paracrine factors secreted by endothelial progenitor cells prevent oxidative stress-induced apoptosis of mature endothelial cells. Atherosclerosis..

[46] Zhou X, Stuart A, Dettin LE, Rodriguez G, Hoel B, Gallicano GI (2004). Desmoplakin is required for microvascular tube formation in culture. J Cell Sci.

[47] Zhang J, Li L, Zhang Q, Yang X, Zhang C, Zhang X (2019). Phosphorylation of microtubule- associated protein 4 promotes hypoxic endothelial cell migration and proliferation. Front Pharmacol.

[48] Sangwung P, Zhou G, Lu Y, Liao X, Wang B, Mutchler SM (2017). Regulation of endothelial hemoglobin alpha expression by Kruppel-like factors. Vasc Med.

[49] Hazarika S, Angelo M, Li Y, Aldrich AJ, Odronic SI, Yan Z (2008). Myocyte specific overexpression of myoglobin impairs angiogenesis after hind-limb ischemia. Arterioscler Thromb Vasc Biol.

[50] Meisner JK, Song J, Annex BH, Price RJ (2013). Myoglobin overexpression inhibits reperfusion in the ischemic mouse hindlimb through impaired angiogenesis but not arteriogenesis. Am J Pathol.

[51] Vinchi F, De Franceschi L, Ghigo A, Townes T, Cimino J, Silengo L (2013). Hemopexin therapy improves cardiovascular function by preventing heme-induced endothelial toxicity in mouse models of hemolytic diseases. Circulation.

[52] Khan AI, Kerfoot SM, Heit B, Liu L, Andonegui G, Ruffell B (2004). Role of CD44 and hyaluronan in neutrophil recruitment. J Immunol.

[53] Nolan S, Dixon R, Norman K, Hellewell P, Ridger V (2008). Nitric oxide regulates neutrophil migration through microparticle formation. Am J Pathol.

[54] Tidball JG (2005). Inflammatory processes in muscle injury and repair. Am J Physiol Regul Integr Comp Physiol.

[55] Smith JA (1994). Neutrophils, host defense, and inflammation: a double-edged sword. J Leukoc Biol.

[56] Xie Q, Klesney-Tait J, Keck K, Parlet C, Borcherding N, Kolb R (2015). Characterization of a novel mouse model with genetic deletion of CD177. Protein Cell.

[57] Sachs UJ, Andrei-Selmer CL, Maniar A, Weiss T, Paddock C, Orlova VV (2007). The neutrophil-specific antigen CD177 is a counter-receptor for platelet endothelial cell adhesion molecule-1 (CD31). J Biol Chem.

[58] Schroll A, Eller K, Feistritzer C, Nairz M, Sonnweber T, Moser PA (2012). Lipocalin-2 ameliorates granulocyte functionality. Eur J Immunol.

[59] Janciauskiene S, Wrenger S, Immenschuh S, Olejnicka B, Greulich T, Welte T (2018). The multifaceted effects of alpha1-antitrypsin on neutrophil functions. Front Pharmacol.

[60] Dunlea DM, Fee LT, McEnery T, McElvaney NG, Reeves EP (2018). The impact of alpha-1 antitrypsin augmentation therapy on neutrophil-driven respiratory disease in deficient individuals. J Inflamm Res.

[61] Lin RZ, Lee CN, Moreno-Luna R, Neumeyer J, Piekarski B, Zhou P, et al. Host non-inflammatory neutrophils mediate the engraftment of bioengineered vascular networks. Nat Biomed Eng. 2017;1:0081. 10.1038/s41551-017-0081 PMC5578427 28868207

[62] Seignez C, Phillipson M (2017). The multitasking neutrophils and their involvement in angiogenesis. Curr Opin Hematol.

[63] Hu C, Yang K, Li M, Huang W, Zhang F, Wang H (2018). Lipocalin 2: a potential therapeutic target for breast cancer metastasis. Onco Targets Ther.

[64] Wu L, Du Y, Lok J, Lo EH, Xing C (2015). Lipocalin-2 enhances angiogenesis in rat brain endothelial cells via reactive oxygen species and iron-dependent mechanisms. J Neurochem.

[65] Hemdahl AL, Gabrielsen A, Zhu C, Eriksson P, Hedin U, Kastrup J (2006). Expression of neutrophil gelatinase-associated lipocalin in atherosclerosis and myocardial infarction. Arterioscler Thromb Vasc Biol.

[66] Abella V, Scotece M, Conde J, Gomez R, Lois A, Pino J (2015). The potential of lipocalin-2/NGAL as biomarker for inflammatory and metabolic diseases. Biomarkers.

[67] Cao G, Savani RC, Fehrenbach M, Lyons C, Zhang L, Coukos G (2006). Involvement of endothelial CD44 during in vivo angiogenesis. Am J Pathol.

[68] Savani RC, Cao G, Pooler PM, Zaman A, Zhou Z, DeLisser HM (2001). Differential involvement of the hyaluronan (HA) receptors CD44 and receptor for HA-mediated motility in endothelial cell function and angiogenesis. J Biol Chem.

[69] Ye Y, Li X, Zhang Y, Shen Z, Yang J (2016). Androgen modulates functions of endothelial progenitor cells through activated Egr1 signaling. Stem Cells Int.

[70] Rahmani M, Wong BW, Ang L, Cheung CC, Carthy JM, Walinski H (2006). Versican: signaling to transcriptional control pathways. Can J Physiol Pharmacol.

[71] Druhan LJ, Lance A, Li S, Price AE, Emerson JT, Baxter SA (2017). Leucine rich alpha-2 glycoprotein: a novel neutrophil granule protein and modulator of myelopoiesis. PLoS One.

[72] Wang X, Abraham S, McKenzie JAG, Jeffs N, Swire M, Tripathi VB (2013). LRG1 promotes angiogenesis by modulating endothelial TGF-beta signalling. Nature.

[73] Song W, Wang X (2015). The role of TGFbeta1 and LRG1 in cardiac remodelling and heart failure. Biophys Rev.

[74] Limbourg A, von Felden J, Jagavelu K, Krishnasamy K, Napp LC, Kapopara PR (2015). MAP-kinase activated protein kinase 2 links endothelial activation and monocyte/macrophage recruitment in arteriogenesis. PLoS One.

[75] Donato R, Cannon BR, Sorci G, Riuzzi F, Hsu K, Weber DJ (2013). Functions of S100 proteins. Curr Mol Med.

[76] Xia C, Braunstein Z, Toomey AC, Zhong J, Rao X (2017). S100 proteins as an important regulator of macrophage inflammation. Front Immunol.

[77] Bao L, Odell AF, Stephen SL, Wheatcroft SB, Walker JH, Ponnambalam S (2012). The S100A6 calcium-binding protein regulates endothelial cell-cycle progression and senescence. FEBS J.

[78] Kim JY, So KJ, Lee S, Park JH (2012). Bcl-rambo induces apoptosis via interaction with the adenine nucleotide translocator. FEBS Lett.

[79] Dong JL, Dong HC, Yang L, Qiu ZW, Liu J, Li H (2018). Upregulation of BAG3 with apoptotic and autophagic activities in maggot extractpromoted rat skin wound healing. Mol Med Rep.

[80] Hassanpour M, Rezabakhsh A, Pezeshkian M, Rahbarghazi R, Nouri M (2018). Distinct role of autophagy on angiogenesis: highlights on the effect of autophagy in endothelial lineage and progenitor cells. Stem Cell Res Ther.

[81] Carrizzo A, Damato A, Ambrosio M, Falco A, Rosati A, Capunzo M (2016). The prosurvival protein BAG3: a new participant in vascular homeostasis. Cell Death Dis.

[82] Kim SW, Kim H, Cho HJ, Lee JU, Levit R, Yoon YS (2010). Human peripheral blood-derived CD31+ cells have robust angiogenic and vasculogenic properties and are effective for treating ischemic vascular disease. J Am Coll Cardiol.

[83] Chavakis E, Dimmeler S (2002). Regulation of endothelial cell survival and apoptosis during angiogenesis. Arterioscler Thromb Vasc Biol.

[84] Dai Q, Thompson MA, Pippen AM, Cherwek H, Taylor DA, Annex BH (2002). Alterations in endothelial cell proliferation and apoptosis contribute to vascular remodeling following hind-limb ischemia in rabbits. Vasc Med.

[85] Vizcaino JA, Csordas A, Del-Toro N, Dianes JA, Griss J, Lavidas I (2016). 2016 update of the PRIDE database and its related tools. Nucleic Acids Res.

