# Supplementary material for: Identification of the initial molecular changes in response to circulating angiogenic cells-mediated therapy in critical limb ischemia
Source: Stem Cell Res Ther. 2020 Mar 6;11:106. doi: 10.1186/s13287-020-01591-0 (PMC7060566; doi:10.1186/s13287-020-01591-0)
Supplement: Supplementary file 1 — Additional file 1: Supplementary Figure S1. CACs administration sites and muscle extraction. Supplementary Figure S2. Characterization of cultured CACs. Supplementary Figure S3. Standard curve for detecting human Alu sequence among the ischemic tissues. Supplementary Figure S4. Graphical correlations between differentially expressed proteins and functions due to ischemia, on day 2. Supplementary Figure S5. Graphical correlations between differentially expressed proteins and functions due to ischemia, on day 4. Supplementary Table 1. Quantitative comparison of proteins differentially expressed by ischemia on days 2 and 4 (SC vs SH). Supplementary Table 2. Quantitative comparison of proteins differentially expressed after CACs treatment on days 2 and 4 (SE vs SC). Supplementary Table 3. Functional classification of proteins differentially expressed by ischemic non-treated mice (SC) vs Shams (SH) on day 2 after femoral ligation. Supplementary Table 4. Functional classification of differentially expressed proteins in ischemic non-treated mice (SC) vs Shams (SH) on day 4 after femoral ligation. Supplementary Table 5. List of antibodies used in this study. Supplementary table 6. Secondary antibodies used in this study. [file 13287_2020_1591_MOESM1_ESM.pdf]

Supplementary figure S1

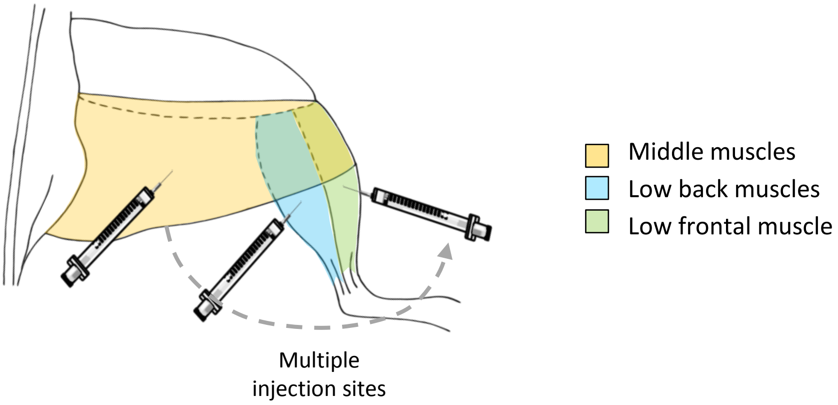

Supplementary Figure S1: CACs administration sites and muscle extraction. Mice received 3-4 intramuscular injections of either 5.10^5 CACs or physiological serum (50 µl in total) in different sites of the muscles surrounding the left femoral artery: low frontal muscles (tibialis), low back (gastrocnemius and soleus) and middle muscles (bicep femoris, adductor and semi-membranous). Low frontal muscles were used for IHC, while low back and middle muscles were kept for Alu-based quantification and proteomic analysis.

Supplementary figure S2

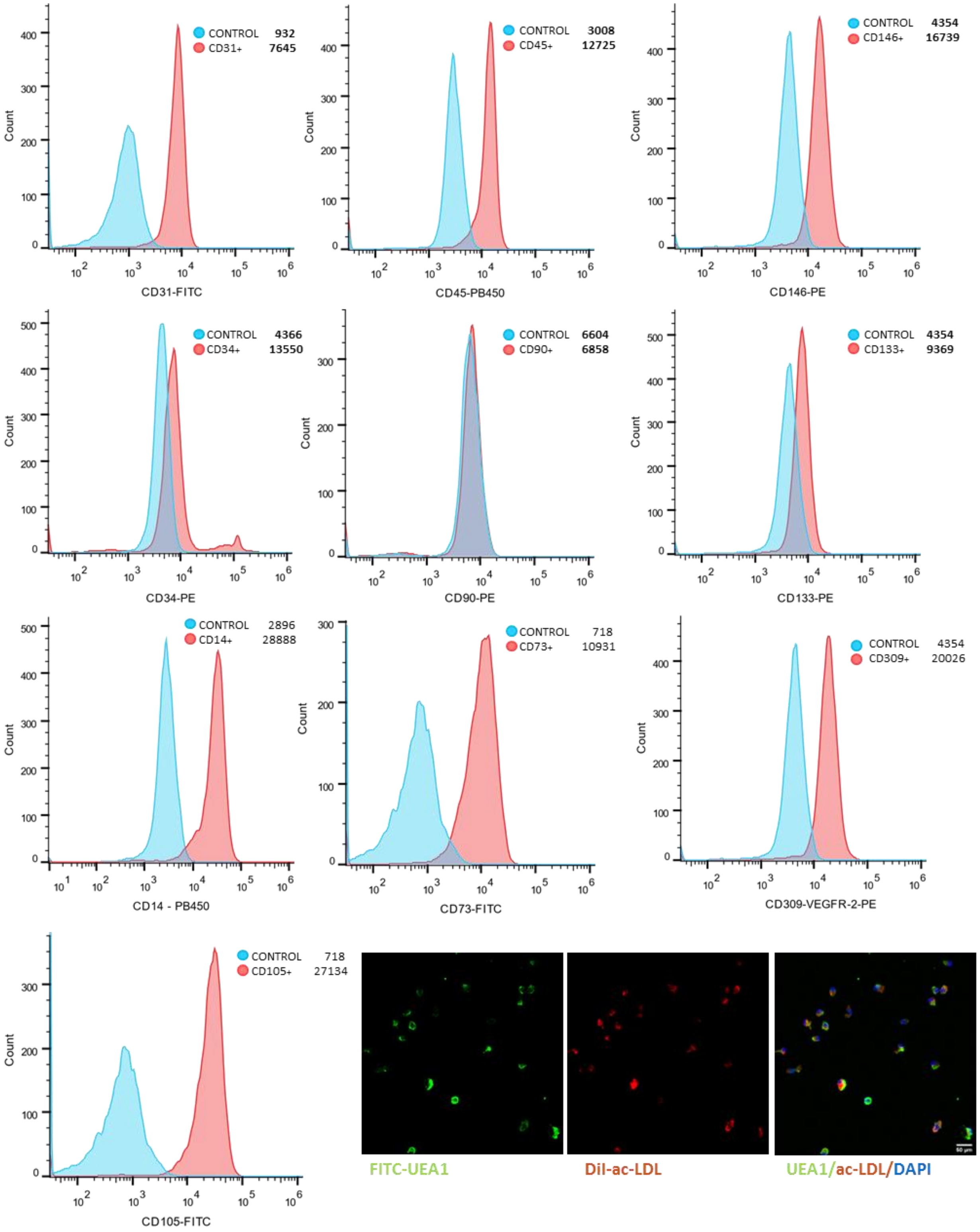

Supplementary Figure S2: Characterization of cultured CACs. Cells were isolated from PBMCs of healthy donors and cultured in fibronectin-coated plates. On day 7, cell identity was confirmed by flow cytometry, analyzing the following markers: CD31, CD34, CD45, CD90, CD73, CD105, CD309 (VEGFR2), CD133, CD146 and CD14. Specific isotype antibodies were used as negative controls. Data were presented as Mean Fluorescence Intensity (MIF), for both, the isotype control (blue) and positive markers analyzed (red). Additionally, CACs identity was also confirmed by IHC, with cells presenting double labeling with UEA1 and Ac-LDL uptake.

Supplementary figure S3

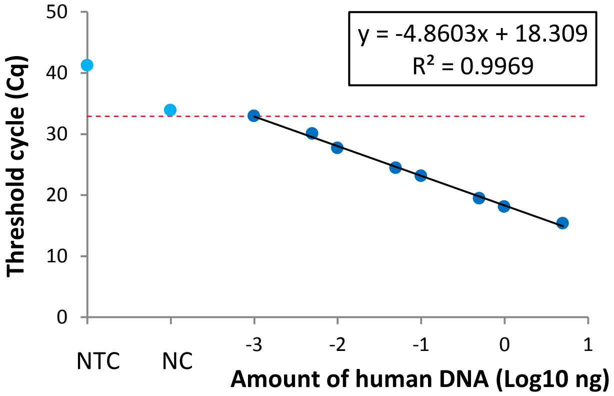

Supplementary Figure S3: Standard curve for detecting human Alu sequence among the mice ischemic tissues. A total of 100 ng of mixed human and mouse genomic DNA (known amounts) were used. Thus, decreasing amounts of human DNA (5, 1, 0.5, 0.1, 0.05, 0.01, 0.005 and 0.001 ng) were mixed with increasing amounts of mouse DNA (95, 99, 99.5, 99.9, 99.95, 99.990 and 99.999 ng). qPCR was performed using triplicates per samples in one run, and the mean Cq values were plotted. Additionally, a negative control (NC), with only mouse genomic DNA, and a non-template control (NTC) were used.

Supplementary figure S4

SC2/SH

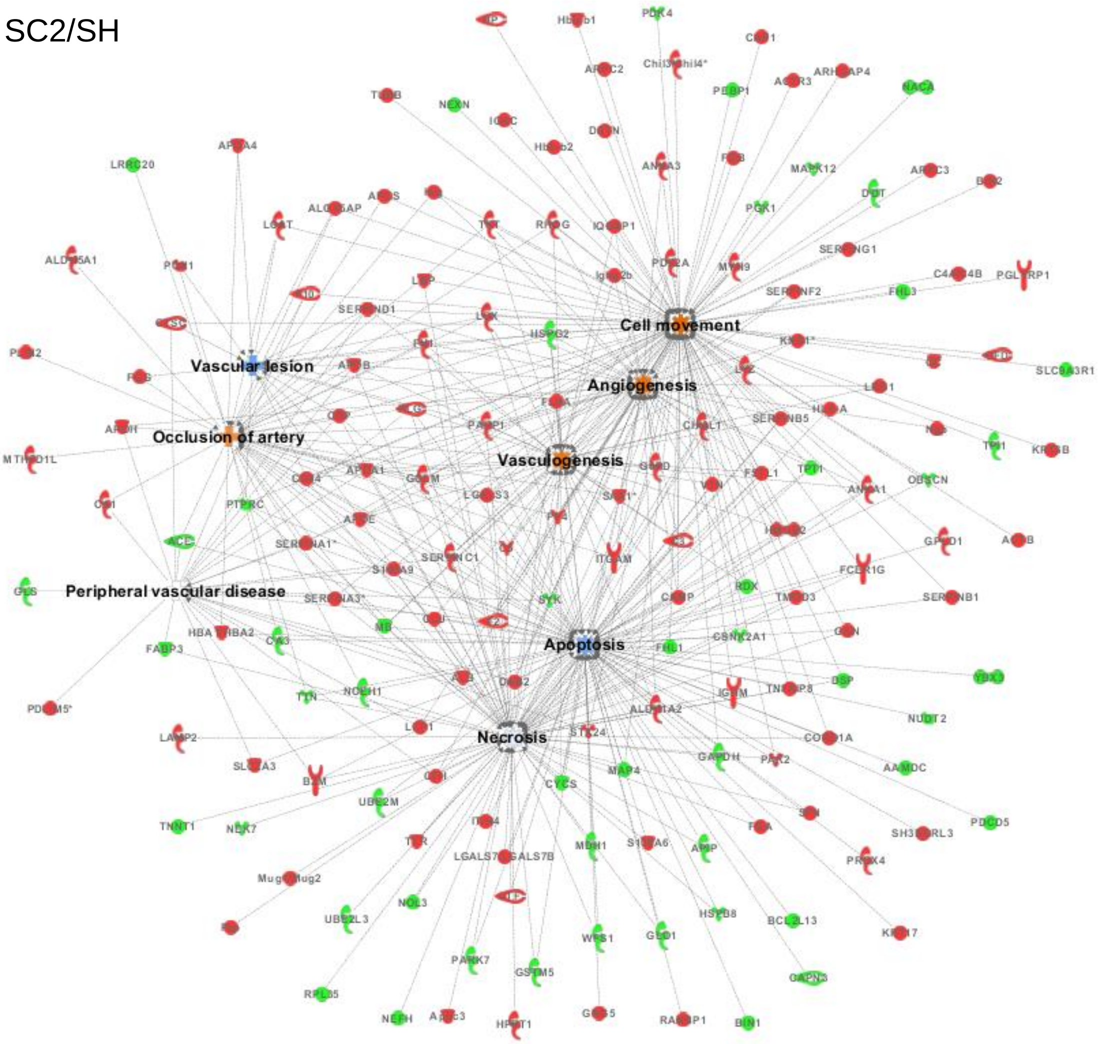

Supplementary Figure S4. Graphical correlations between differentially expressed proteins and functions due to ischemia on day 2. Major functions described for proteins with expression changes in ischemic non-treated mice (SC) on day 2 compared to sham mice (SH): $p<0.05$. Pathways were obtained with the Ingenuity Pathway Analysis Software (IPA). Color legend: increased measurement (●), decreased measurement (●), predicted activation (more ● or less ● confidence) and predicted inhibition (more ● or less ● confidence)

Supplementary figure S5

SC4/SH

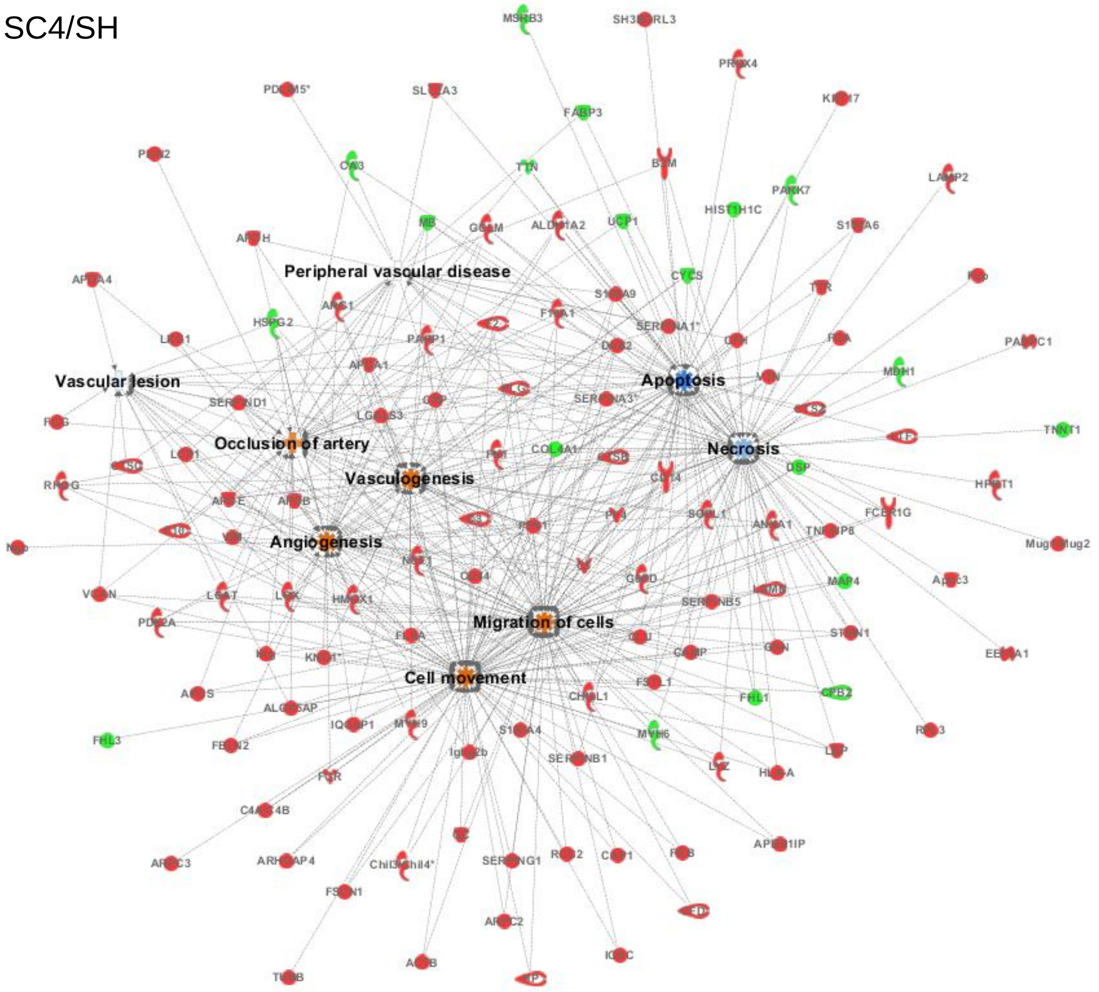

Supplementary Figure S5. Graphical correlations between proteins differentially expressed and functions due to ischemia on day 4. Major functions described for proteins with expression changes in ischemic non-treated mice (SC) on day 4 compared to sham mice (SH): $p<0.05$. Pathways were obtained with the IPA software. Color legend: increased measurement (●), decreased measurement (●), predicted activation (more ● or less ● confidence) and predicted inhibition (more ● or less ● confidence)

Supplementary Table 1. Quantitative comparison of proteins differentially expressed by ischemia on days 2 and 4 (SC *vs* SH). The table includes (from left to right) Uniprot accession number, protein description, abundance ratio on days 2 and 4 (considering up-regulated ratio>1.5 and down-regulated ratio<0.6), P-value on days 2 and 4 (considering P-value<0.05 as differentially significant) and biological processes in which proteins are involved: Response to stimulus (RS), cell organization and biogenesis (COB), metabolic process (MP), regulation of biological process (RBP), transport (T), cell differentiation (CDif), cell death (CDe), cellular homeostasis (CH), coagulation (C), defense response (DR), cellular component movement (CCM), development (D), cell proliferation (CP), cell communication (CC), cell growth (CG) and cell division (CDiv).

		Abundance ratio		P-value		
**Accession**	**Description**	**SC2/SH**	**SC4/SH**	**SC2/SH**	**SC4/SH**	**Biological Process**
A0A0B4J1H7	Immunoglobulin kappa variable 1-135 (Fragment)	100.00000	100.00000	0.00000	0.00000	RS
P30355	Arachidonate 5-lipoxygenase-activating protein	8.47458	15.38462	0.00000	0.00001	COB; MP; RBP; RS
E9PV24	Fibrinogen alpha chain	8.92857	12.50000	0.00000	0.00004	COB; C; DR; MP; RBP; RS
Q62356	Follistatin-related protein 1	4.32900	8.26446	0.00040	0.00069	RS; T
Q91X72	Hemopexin	7.93651	10.10101	0.00000	0.00018	CH; MP; RBP; T
P06728	Apolipoprotein A-IV	9.09091	5.84795	0.00000	0.00553	COB; DR; MP; RBP; RS; T
P07759	Serine protease inhibitor A3K	5.61798	5.88235	0.00003	0.00534	RBP; RS
Q01339	Beta-2-glycoprotein 1	13.51351	23.80952	0.00000	0.00000	C; MP; RBP; RS; T
Q80Y14	Glutaredoxin-related protein 5, mitochondrial	90.90909	40.00000	0.00000	0.00000	CH; MP; RBP
P29788	Vitronectin	5.26316	6.80272	0.00005	0.00235	CDif; COB; RBP; RS; T
Q00897	Alpha-1-antitrypsin 1-4	2.77778	7.29927	0.01423	0.00154	RBP; RS
P31725	Protein S100-A9	90.90909	38.46154	0.00000	0.00000	CDe; COB; CCM; DR; MP; RBP; RS
P07309	Transthyretin	13.88889	10.98901	0.00000	0.00010	MP; RBP; T
Q3UER8	Fibrinogen gamma chain	11.76471	22.72727	0.00000	0.00000	COB; C; DR; MP; RBP; RS; T
Q61838	Pregnancy zone protein	4.25532	4.65116	0.00045	0.01817	RBP
Q00623	Apolipoprotein A-I	4.65116	6.06061	0.00019	0.00460	COB; CP; CCM; MP; RBP; RS; T

P08226	Apolipoprotein E	3.33333	5.49451	0.00373	0.00792	COB; CH; MP; RBP; RS; T
P19221	Prothrombin	5.05051	6.28931	0.00008	0.00370	CH; C; DR; MP; RBP; RS
Q3U3V1	Coagulation factor X	100.00000	100.00000	0.00000	0.00000	C; MP; RBP
Q6S9I0	Kininogen 2	100.00000	100.00000	0.00000	0.00000	CH; RBP
P39039	Mannose-binding protein A	3.18471	5.29101	0.00521	0.00953	COB; DR; MP; RBP; RS
P20491	High affinity immunoglobulin epsilon receptor subunit gamma	3.66300	4.71698	0.00189	0.01698	CDif; COB; CCM; DR; MP; RBP; RS; T
Q8BTM8	Filamin-A	2.42718	3.95257	0.03448	0.03793	COB; CCM; MP; RBP; RS; T
P01029	Complement C4-B	3.25733	3.98406	0.00442	0.03688	DR; MP; RBP; RS
Q99K85	Phosphoserine aminotransferase	3.27869	8.40336	0.00487	0.00062	MP
P32037	Solute carrier family 2, facilitated glucose transporter member 3	100.00000	100.00000	0.00000	0.00000	T
P08905	Lysozyme C-2	6.94444	16.39344	0.00000	0.00000	DR; MP; RS
A2AL50	Alkylglycerone-phosphate synthase	100.00000	100.00000	0.00000	0.00000	MP
Q9CQF3	Cleavage and polyadenylation specificity factor subunit 5	100.00000	100.00000	0.00000	0.00000	COB; MP
P70124	Serpin B5	100.00000	100.00000	0.00000	0.00000	COB; RBP
Q80Z68	Rho GTPase activating protein 4, isoform CRA_f	100.00000	100.00000	0.00000	0.00000	RBP; RS
Q91Z98	Chitinase-like protein 4	8.13008	5.88235	0.00000	0.00950	DR; MP; RS
P16301	Phosphatidylcholine-sterol acyltransferase	100.00000	100.00000	0.00000	0.00000	COB; MP; RBP; RS; T
Q9D7S7	60S ribosomal protein L22-like 1	100.00000	100.00000	0.00000	0.00000	MP
P01807	Ig heavy chain V region X44	3.98406	14.49275	0.00082	0.00001	
Q62148	Retinal dehydrogenase 2	100.00000	100.00000	0.00000	0.00000	CDif; MP; RBP; RS
A0A0B4J1I0	Immunoglobulin kappa variable 1-110	100.00000	100.00000	0.00000	0.00000	RS

	(Fragment)					
O09172	Glutamate--cysteine ligase regulatory subunit	100.00000	100.00000	0.00000	0.00000	COB; CH; MP; RBP; RS
F7D3W5	Phosphodiesterase	100.00000	100.00000	0.00000	0.00000	RBP; RS
A0A0G2JEA5	Predicted gene 43738	100.00000	100.00000	0.00000	0.00000	MP
Q8CI51	PDZ and LIM domain protein 5	100.00000	100.00000	0.00000	0.00000	CG; RBP
A0A2I3BRQ1	Expressed sequence AI182371	100.00000	100.00000	0.00000	0.00000	
D3Z312	Tumor necrosis factor alpha-induced protein 8	100.00000	100.00000	0.00000	0.00000	MP; RBP
Q02105	Complement C1q subcomponent subunit C	100.00000	100.00000	0.00000	0.00000	DR; MP; RBP; RS
Q8K0E8	Fibrinogen beta chain	15.62500	31.25000	0.00000	0.00000	COB; C; DR; MP; RBP; RS
Q8VDD5	Myosin-9	2.83286	5.18135	0.01245	0.01074	CDif; COB; CCM; MP; RBP; T
Q9CPW4	Actin-related protein 2/3 complex subunit 5	3.44828	7.24638	0.00277	0.00159	COB; CCM; RBP
A2AMW0	Capping protein (Actin filament) muscle Z-line, beta, isoform CRA_a	100.00000	100.00000	0.00000	0.00000	COB; RBP
Q91Z25	Actin-related protein 2/3 complex subunit	2.39808	4.44444	0.03738	0.02268	COB; RBP
Q9CVB6	Actin-related protein 2/3 complex subunit 2	2.59740	4.92611	0.02266	0.01369	COB; RBP; RS
Q9Z126	Platelet factor 4	5.34759	28.57143	0.00004	0.00000	COB; CCM; DR; RBP; RS
A0A0R4J038	Kininogen-1	6.99301	10.00000	0.00000	0.00019	CH; C; DR; RBP; RS
B8JJN0	Predicted gene 20547	8.33333	9.70874	0.00000	0.00023	CP; DR; MP; RBP; RS
Q61704	Inter-alpha-trypsin inhibitor heavy chain H3	3.25733	6.28931	0.00442	0.00374	MP; RBP
P21614	Vitamin D-binding protein	10.30928	14.49275	0.00000	0.00001	MP; T
P12246	Serum amyloid P-component	8.92857	9.80392	0.00000	0.00022	COB; DR; RBP; RS
P97290	Plasma protease C1 inhibitor	5.98802	10.00000	0.00001	0.00019	C; DR; MP; RBP; RS

Q60590	Alpha-1-acid glycoprotein 1	4.38596	5.98802	0.00033	0.00484	DR; RBP; RS; T
P03953	Complement factor D	3.18471	4.09836	0.00525	0.03268	DR; MP; RBP; RS
E0CZ58	Proteoglycan 4	2.41546	3.86100	0.03573	0.04198	CP; RBP; RS; T
P01887	Beta-2-microglobulin	3.74532	5.15464	0.00140	0.01098	CDif; DR; MP; RBP; RS; T
P97821	Dipeptidyl peptidase 1	100.00000	100.00000	0.00000	0.00000	CDe; MP; RBP; RS
Q9Z1Q5	Chloride intracellular channel protein 1	2.81690	6.28931	0.01286	0.00367	MP; RBP; T
P45377	Aldose reductase-related protein 2	100.00000	100.00000	0.00000	0.00000	MP
P14069	Protein S100-A6	5.74713	7.19424	0.00002	0.00172	T
Q8BWM3	MCG130173	100.00000	100.00000	0.00000	0.00000	RBP
P59999	Actin-related protein 2/3 complex subunit 4	2.64550	4.32900	0.01978	0.02534	COB; RBP
A0A0A0MQA3	Alpha-1-antitrypsin 1-1	4.23729	4.80769	0.00047	0.01528	MP; RS
A0A1W2P6X3	Formin-like protein 1	100.00000	100.00000	0.00000	0.00000	COB; CCM; RBP
G3X8Q5	Ceruloplasmin	5.71429	7.35294	0.00002	0.00150	CH; MP; RS; T
O08692	Neutrophilic granule protein	22.22222	12.19512	0.00000	0.00004	DR; RBP; RS
O89020	Afamin	6.75676	5.81395	0.00000	0.00582	T
P50543	Protein S100-A11	7.93651	8.47458	0.00000	0.00060	RBP
Q91XL1	Leucine-rich HEV glycoprotein	12.65823	7.87402	0.00000	0.00098	CDif; RBP
P29699	Alpha-2-HS-glycoprotein	5.31915	5.78035	0.00005	0.00586	COB; DR; RBP; RS
O35744	Chitinase-like protein 3	3.28947	4.38596	0.00406	0.02373	DR; MP; RS
Q91VW3	SH3 domain-binding glutamic acid-rich-like protein 3	3.59712	5.74713	0.00200	0.00622	CH; MP; RBP
P14847	C-reactive protein	100.00000	100.00000	0.00000	0.00000	COB; DR; MP; RBP; RS

P01867	Ig gamma-2B chain C region	100.00000	100.00000	0.00000	0.00000	COB; DR; MP; RBP; RS
A0A1Y7VJN6	Immunoglobulin heavy constant gamma 3 (Fragment)	3.48432	6.25000	0.00256	0.00388	
P16110	Galectin-3	4.97512	14.70588	0.00009	0.00001	CDif; COB; CCM; DR; MP; RBP; RS
A0A087WR50	Fibronectin	5.58659	11.36364	0.00003	0.00007	CDif; COB; CCM; DR; MP; RBP; RS
P28665	Murinoglobulin-1	5.31915	8.69565	0.00005	0.00050	RBP
Q00612	Glucose-6-phosphate 1-dehydrogenase X	3.10559	4.90196	0.00638	0.01393	MP; RBP; RS
P20918	Plasminogen	12.50000	20.83333	0.00000	0.00000	CDif; COB; CCM; CH; C; MP; RBP
E9Q8I0	Complement factor H	5.58659	6.89655	0.00003	0.00218	DR; MP; RBP; RS
P60710	Actin, cytoplasmic 1	2.58398	5.29101	0.02345	0.00958	COB; RS
P51437	Cathelicidin antimicrobial peptide	14.28571	8.77193	0.00000	0.00047	DR; RBP; RS
P01027	Complement C3	7.57576	8.06452	0.00000	0.00083	COB; C; DR; MP; RBP; RS
Q9JKF1	Ras GTPase-activating-like protein IQGAP1	2.48756	4.09836	0.02978	0.03276	CG; RBP; RS
Q61233	Plastin-2	6.57895	10.41667	0.00000	0.00015	COB; CCM; RBP; RS
P10107	Annexin A1	7.51880	9.17431	0.00000	0.00036	CDif; COB; CCM; DR; MP; RBP; RS; T
Q61646	Haptoglobin	11.76471	11.11111	0.00000	0.00009	DR; MP; RBP; RS
Q91WP6	Serine protease inhibitor A3N	15.87302	13.51351	0.00000	0.00002	DR; RBP; RS
P13020	Gelsolin	4.34783	4.71698	0.00036	0.01692	CDe; COB; MP; RBP; RS; T
P40124	Adenylyl cyclase-associated protein 1	3.43643	4.60829	0.00289	0.01909	COB; CCM; RBP; RS; T
A0A0R4J039	Histidine-rich glycoprotein	11.62791	16.66667	0.00000	0.00000	C; DR; RBP; RS; T
P22599	Alpha-1-antitrypsin 1-2	5.71429	6.71141	0.00002	0.00251	DR; MP; RBP; RS
E9Q414	Apolipoprotein B-100	100.00000	100.00000	0.00000	0.00000	COB; CCM; MP; RBP; RS; T

P31532	Serum amyloid A-4 protein	7.75194	14.49275	0.00000	0.00001	CCM; DR; RS
E9Q035	Predicted gene 20425	5.12821	5.07614	0.00007	0.01174	RBP; RS
P70698	CTP synthase 1	100.00000	100.00000	0.00000	0.00000	CP; MP
Q61362	Chitinase-3-like protein 1	100.00000	100.00000	0.00000	0.00000	CDe; DR; MP; RBP; RS; T
P34928	Apolipoprotein C-I	8.33333	13.88889	0.00000	0.00002	COB; MP; RBP; T
E9QP56	Apolipoprotein C-III	10.63830	16.39344	0.00000	0.00000	COB; CH; MP; RBP; RS; T
E9Q6I2	Interleukin-1 receptor accessory protein	6.57895	5.95238	0.00000	0.00510	DR; MP; RBP; RS; T
Q9CYL5	Golgi-associated plant pathogenesis-related protein 1	100.00000	100.00000	0.00000	0.00000	RBP
F8WHQ1	Tumor protein D52	100.00000	100.00000	0.00000	0.00000	CDif; RBP
A0A0R4J0M4	Apolipoprotein F	100.00000	100.00000	0.00000	0.00000	MP; T
P49222	Erythrocyte membrane protein band 4.2	100.00000	100.00000	0.00000	0.00000	COB; MP; RBP
P23953	Carboxylesterase 1C	8.54701	5.49451	0.00000	0.00780	MP
P06684	Complement C5	2.93255	4.83092	0.00973	0.01509	DR; MP; RBP; RS
P00493	Hypoxanthine-guanine phosphoribosyltransferase	2.73224	5.18135	0.01593	0.01067	CDif; COB; CP; MP; RBP; RS
Q8BVE3	V-type proton ATPase subunit H	4.03226	4.60829	0.00074	0.01914	CH; T
P51910	Apolipoprotein D	2.55754	3.75940	0.02500	0.04720	COB; MP; RBP; RS; T
Q2M2N2	Speckle-type POZ protein-like	11.23596	11.62791	0.00000	0.00006	MP; RBP
Q9D154	Leukocyte elastase inhibitor A	4.13223	4.83092	0.00057	0.01512	RBP
Q07456	Protein AMBP	9.43396	9.00901	0.00000	0.00039	MP; RBP
Q9JM76	Actin-related protein 2/3 complex subunit 3	2.48756	3.83142	0.02991	0.04365	COB; RBP; RS
Q05020	Apolipoprotein C-II	4.38596	5.26316	0.00033	0.00982	MP; RBP; T

P54116	Erythrocyte band 7 integral membrane protein	3.75940	5.68182	0.00261	0.01133	COB; RBP
A0A1W2P7Q9	Myosin light polypeptide 6	2.35294	4.06504	0.04162	0.03383	CCM
P84096	Rho-related GTP-binding protein RhoG	2.39808	5.68182	0.03724	0.00651	COB; CCM; RBP; RS
O08807	Peroxiredoxin-4	100.00000	100.00000	0.00000	0.00000	COB; CH; MP; RBP
P50172	Corticosteroid 11-beta-dehydrogenase isozyme 1	2.46914	4.42478	0.04740	0.04157	MP; RBP
Q06890	Clusterin	3.87597	5.78035	0.00103	0.00600	COB; CP; MP; RBP; RS; T
P26262	Plasma kallikrein	3.50877	6.75676	0.00241	0.00246	C; DR; MP; RBP; RS
G3UXA6	Polypyrimidine tract-binding protein 3	2.67380	5.98802	0.02118	0.00498	CDif; MP; RBP
Q61805	Lipopolysaccharide-binding protein	2.81690	4.73934	0.01307	0.01639	CCM; DR; RBP; RS; T
P99024	Tubulin beta-5 chain	2.53165	6.02410	0.02658	0.00481	COB
P01837	Immunoglobulin kappa constant	3.08642	7.40741	0.00669	0.00138	
P08071	Lactotransferrin	9.09091	6.36943	0.00000	0.00341	DR; MP; RBP; RS; T
P49182	Heparin cofactor 2	3.30033	5.15464	0.00402	0.01087	C; RBP
P43883	Perilipin-2	11.90476	7.69231	0.00000	0.00114	RS; T
P46664	Adenylosuccinate synthetase isozyme 2	4.29185	8.40336	0.00047	0.00063	MP; RS
A0A0R4J104	Disabled homolog 2	5.81395	9.09091	0.00004	0.00037	CDe; CDif; COB; D; RBP; RS; T
P28301	Protein-lysine 6-oxidase	3.26797	9.25926	0.00482	0.00033	COB; MP; RS
P07361	Alpha-1-acid glycoprotein 2	14.92537	16.39344	0.00000	0.00000	DR; RBP; RS; T
P47968	Ribose-5-phosphate isomerase	3.66300	6.13497	0.00183	0.00432	MP
Q8BFZ3	Beta-actin-like protein 2	8.06452	7.69231	0.00000	0.00173	
Q9QWL7	Keratin, type I cytoskeletal 17	2.65252	4.54545	0.01971	0.02042	COB; RBP; RS

P01786	Ig heavy chain V region MOPC 47A	3.92157	5.40541	0.00162	0.00855	
P01898	H-2 class I histocompatibility antigen, Q10 alpha chain	2.31481	4.38596	0.04637	0.02404	
P47753	F-actin-capping protein subunit alpha-1	2.58398	3.77358	0.02339	0.04657	COB; RBP
A2APM2	CD44 antigen	4.56621	4.31034	0.00023	0.02590	COB; CCM; MP; RBP; RS
P17047	Lysosome-associated membrane glycoprotein 2	3.12500	5.88235	0.00607	0.00542	CC; COB; CH; MP; RBP; RS; T
Q3UV17	Keratin, type II cytoskeletal 2 oral	3.93701	7.63359	0.00090	0.00117	
Q7TPV4	Myb-binding protein 1A	2.97619	4.90196	0.00870	0.01391	CC; CDif; MP; RBP; RS; T
Q921K2	Poly [ADP-ribose] polymerase	100.00000	100.00000	0.00000	0.00000	CDe; COB; MP; RBP; RS
Q3THE2	Myosin regulatory light chain 12B	2.13675	3.92157	0.07232	0.03959	RBP
Q8VIJ6	Splicing factor, proline- and glutamine-rich	2.27790	4.65116	0.05047	0.01798	COB; DR; MP; RBP; RS
P01865	Ig gamma-2A chain C region, membrane-bound form	1.95695	14.08451	0.13966	0.00001	COB; DR; MP; RBP; RS; T
Q61702	Inter-alpha-trypsin inhibitor heavy chain H1	2.26244	4.76190	0.05273	0.01613	MP; RBP
Q62059	Versican core protein	1.76678	4.48430	0.18606	0.02149	CDif; CCM; RBP
P10126	Elongation factor 1-alpha 1	2.12314	5.68182	0.07482	0.00648	COB; MP; RBP; RS
P62962	Profilin-1	2.20751	3.95257	0.06034	0.03793	COB; RBP; RS
Q8BH61	Coagulation factor XIII A chain	1.60514	5.05051	0.27854	0.01200	C; MP; RS
P20152	Vimentin	1.64745	4.34783	0.25105	0.02485	CDif; COB; MP; RBP; RS
P14901	Heme oxygenase 1	2.22222	7.14286	0.05854	0.00174	CC; CDe; COB; CH; MP; RBP; RS
P27659	60S ribosomal protein L3	1.59744	5.00000	0.28448	0.01277	COB; MP; RS
Q8BK67	Protein RCC2	2.05761	4.34783	0.10964	0.02470	CDiv; COB; RBP; RS
Q922I7	MCG13402, isoform CRA_c	1.61551	4.44444	0.27229	0.02235	MP; RBP

P29341	Polyadenylate-binding protein 1	1.52672	3.95257	0.33945	0.03837	MP; RBP
Q6IRU2	Tropomyosin alpha-4 chain	2.17865	4.46429	0.06540	0.02209	CDif; COB
Q9R0E2	Procollagen-lysine,2-oxoglutarate 5-dioxygenase 1	1.77620	5.29101	0.21181	0.00963	MP; RS
P14234	Tyrosine-protein kinase Fgr	2.17391	4.73934	0.06604	0.01653	CDif; DR; MP; RBP; RS
P43275	Histone H1.1	2.17865	5.18135	0.06529	0.01055	COB; RBP
Q64674	Spermidine synthase	1.74520	4.40529	0.19635	0.02349	MP
Q9WUU7	Cathepsin Z	2.05339	5.64972	0.08954	0.00678	MP; RBP
E9PW66	Nucleosome assembly protein 1-like 1	1.98413	4.09836	0.10750	0.03269	COB
O89086	RNA-binding protein 3	1.53846	3.77358	0.32806	0.04673	MP; RBP; RS
Q922Q8	Leucine-rich repeat-containing protein 59	1.52672	4.14938	0.33793	0.03084	
Q8R0X7	Sphingosine-1-phosphate lyase 1	1.66667	4.54545	0.24695	0.02026	CDe; CDif; CCM; MP; RBP; RS
P54227	Stathmin	1.75747	4.62963	0.19307	0.01836	CDif; COB; D; RBP; RS
P10810	Monocyte differentiation antigen CD14	1.68350	4.97512	0.24376	0.02313	DR; RBP; RS; T
Q9JM83	Calmodulin-4	8.13008	2.37530	0.00000	0.34156	RBP; RS
Q3U1U4	Integrin alpha-M	2.87356	3.38983	0.01134	0.07210	COB; CP; CCM; MP; RBP; RS
P32261	Antithrombin-III	6.13497	1.68067	0.00001	0.58419	C; DR; RBP; RS; T
O88593	Peptidoglycan recognition protein 1	2.70270	3.64964	0.02887	0.09224	CDe; DR; MP; RBP; RS
P05366	Serum amyloid A-1 protein	11.76471	2.65957	0.00000	0.17257	CCM; DR; MP; RS
P05367	Serum amyloid A-2 protein	21.27660	3.67647	0.00000	0.07484	CCM; DR; RS
D3Z5G7	Carboxylic ester hydrolase	4.06504	3.17460	0.00067	0.09274	MP
Q8BND5	Sulfhydryl oxidase 1	4.23729	3.55872	0.00046	0.05974	CH; MP; RBP

Q9QXC1	Fetuin-B	5.58659	3.08642	0.00003	0.10233	RBP
P07724	Serum albumin	5.29101	3.22581	0.00005	0.08750	CC; RBP; RS; T
P04919	Band 3 anion transport protein	3.08642	3.17460	0.00666	0.09237	COB; CH; RBP; RS; T
P02088	Hemoglobin subunit beta-1	4.13223	3.08642	0.00058	0.10240	RBP; T
P02089	Hemoglobin subunit beta-2	3.62319	3.06748	0.00189	0.10455	RBP; RS; T
E9PVD2	Inter alpha-trypsin inhibitor, heavy chain 4	4.32900	3.46021	0.00037	0.06651	DR; MP; RBP; RS
P40142	Transketolase	2.70270	3.18471	0.01739	0.09195	MP; RBP
P01872	Immunoglobulin heavy constant mu	3.49650	3.11526	0.00252	0.09962	COB; DR; MP; RBP; RS; T
P01942	Hemoglobin subunit alpha	3.00300	2.23714	0.00812	0.29059	RS; T
O35639	Annexin A3	2.53807	2.73973	0.02623	0.15565	DR; RBP; RS; T
P46412	Glutathione peroxidase 3	2.61780	3.47222	0.02134	0.06545	COB; MP; RS
Q99JY9	Actin-related protein 3	2.47525	3.70370	0.03041	0.05035	CDif; CDiv; COB; CCM; RBP; RS
Q3UHZ7	UDP-N-acetylhexosamine pyrophosphorylase	3.16456	3.34448	0.00974	0.10584	MP
P30681	High mobility group protein B2	2.42131	2.00000	0.03521	0.38995	COB; CCM; DR; MP; RBP; RS
P62806	Histone H4	2.50627	3.03951	0.02840	0.10920	COB; MP; RBP
Q9R0P5	Destrin	2.58398	3.41297	0.02345	0.06991	COB; CCM; RBP
O89053	Coronin-1A	2.70270	2.34192	0.01716	0.25456	COB; CCM; RBP; RS; T
Q61247	Alpha-2-antiplasmin	2.46305	2.40964	0.03160	0.23464	COB; DR; RBP; RS
Q497J0	MCG130175, isoform CRA_b	4.50450	2.94118	0.00025	0.12234	RBP
Q9Z1F9	SUMO-activating enzyme subunit 2	2.91545	2.07469	0.01013	0.41286	MP
P52430	Serum paraoxonase/arylesterase 1	3.17460	3.50877	0.00543	0.06294	MP; RBP; RS
Q80SZ7	Guanine nucleotide-binding protein	3.69004	3.31126	0.00159	0.07881	RBP; RS

	G(I)/G(S)/G(O) subunit gamma-5					
P34022	Ran-specific GTPase-activating protein	2.84900	3.57143	0.01197	0.05841	COB; MP; RBP; RS; T
P08032	Spectrin alpha chain, erythrocytic 1	2.69542	1.90114	0.01752	0.44285	COB; MP; RBP
Q06770	Corticosteroid-binding globulin	4.21941	2.30947	0.00049	0.26564	MP; RBP; T
P13634	Carbonic anhydrase 1	2.73973	1.91939	0.01561	0.43210	MP
Q6P069	Sorcin	2.47525	3.21543	0.03050	0.08856	MP; RBP; T
Q9CPX8	Cytochrome b-c1 complex subunit 10	2.85714	3.50877	0.02121	0.10763	MP; T
E9PVA8	eIF-2-alpha kinase activator GCN1	3.03030	3.10559	0.01362	0.14444	CC; RBP; RS
A0A1L1SRX2	AMP deaminase	4.56621	2.79330	0.00027	0.18312	MP
S4R270	Bridging integrator 2	2.63158	2.81690	0.02886	0.15432	COB; CCM; RS
Q9JHJ0	Tropomodulin-3	2.56410	3.32226	0.03081	0.10538	COB; RBP
Q8CIN4	Serine/threonine-protein kinase PAK 2	2.53807	3.41297	0.02629	0.07060	CDe; COB; CCM; MP; RBP; RS
Q99KH8	Serine/threonine-protein kinase 24	2.53165	2.62467	0.04217	0.23102	CC; CDe; COB; MP; RBP; RS
Q9ERE7	LRP chaperone MESD	3.47222	2.45700	0.00264	0.23688	MP; RBP; RS; T
Q3V3R1	Monofunctional C1-tetrahydrofolate synthase, mitochondrial	2.94118	2.40964	0.00962	0.23399	MP
A0A0R4IZY0	Thimet oligopeptidase	4.16667	1.61290	0.00054	0.63568	MP; RBP; RS
Q8VCU2	Glycosylphosphatidylinositol specific phospholipase D1	2.29358	2.49377	0.04834	0.21132	CDif; CCM; MP; RBP; RS; T
Q8VC88	Grancalcin		100.00000		0.00000	MP
Q6ZQM8	UDP-glucuronosyltransferase 1-7C	0.58858	4.21941	0.16649	0.02839	MP
Q8BJY1	26S proteasome non-ATPase regulatory subunit 5		100.00000		0.00000	COB
Q9ES74	Serine/threonine-protein kinase Nek7	0.26824	1.70068	0.00080	0.56993	MP; RBP

Q6ZWV7	60S ribosomal protein L35	0.45475	1.56006	0.03917	0.67892	MP; RS
P37889	Fibulin-2	1.29199	8.47458	0.63540	0.00060	COB; RBP
P35173	Stefin-3	0.98039	3.78788	0.88367	0.04596	RBP
F8WH69	Neutrophil cytosol factor 1	1.45773	4.42478	0.39934	0.02290	CDe; COB; CP; DR; MP; RBP; RS; T
Q61176	Arginase-1	1.30890	5.00000	0.56645	0.01273	COB; DR; MP; RBP; RS
O89017	Legumain	1.36986	5.37634	0.50006	0.00882	MP; RBP; RS
E9Q0F0	Keratin 78	1.25786	6.06061	0.64019	0.00457	COB; CCM; MP; RBP; T
P61924	Coatomer subunit zeta-1	0.87260	4.14938	0.65356	0.03076	T
P10605	Cathepsin B	1.13122	3.96825	0.83383	0.03749	CDif; MP; RBP; RS
P07091	Protein S100-A4	1.28866	4.25532	0.59398	0.02749	RBP
Q3TWW8	Serine/arginine-rich splicing factor 6	1.48148	5.05051	0.37699	0.01198	COB; MP; RBP; RS
Q61553	Fascin	1.49477	3.77358	0.36602	0.04636	COB; CCM; RBP
Q8R5A3	Amyloid beta A4 precursor protein-binding family B member 1-interacting protein	1.18203	4.71698	0.79261	0.01818	RBP; RS
Q9Z331	Keratin, type II cytoskeletal 6B	100.00000		0.00000		COB; MP
P00848	ATP synthase subunit a	100.00000		0.00000		MP; RS; T
P20060	Beta-hexosaminidase subunit beta	1.95313		0.12545		COB; CCM; CH; MP; RBP; reproduction; RS
P70195	Proteasome subunit beta type-7	3.31126	1.31062	0.00392	0.91686	MP
O70456	14-3-3 protein sigma	2.47525	0.81500	0.03074	0.39282	CDif; COB; RBP; RS
A0A1B0GRV0	3'(2'),5'-bisphosphate nucleotidase 1	3.03030	1.32450	0.00770	0.90118	MP
Q9CRB1	Galectin	2.63852	0.68823	0.02026	0.29668	CDe
A0A140T8T7	Collagen alpha-5(VI) chain	6.13497	1.15075	0.00002	0.78315	

Q8BH35	Complement component C8 beta chain	3.35570	0.76982	0.00350	0.39842	DR; MP; RBP; RS
Q8BWF0	Succinate-semialdehyde dehydrogenase, mitochondrial	2.88184	1.12867	0.01695	0.75927	COB; MP
P50462	Cysteine and glycine-rich protein 3	0.18674	0.27397	0.00002	0.00723	CDif; COB; CH; D; MP; RBP; RS
E9Q3E2	Synaptopodin	0.22999	0.33841	0.00017	0.02093	COB; RBP; RS
Q9QZ25	Vascular non-inflammatory molecule 3	0.12265	0.23079	0.00000	0.00277	MP
Q9WUZ5	Troponin I, slow skeletal muscle	0.34626	0.35236	0.00611	0.02525	RBP
E9Q8K5	Titin	0.20678	0.24219	0.00006	0.00367	MP
O88346	Troponin T, slow skeletal muscle	0.46083	0.38610	0.04237	0.03807	RBP
Q9JJW5	Myozenin-2	0.12674	0.28785	0.00000	0.00938	COB
P32848	Parvalbumin alpha	0.13432	0.35613	0.00000	0.02652	CH
Q9CRA2	PDZ and LIM domain protein 5	0.12344	0.24492	0.00000	0.00390	CG; RBP
Q91YE8	Synaptopodin-2	0.18839	0.25947	0.00002	0.00539	COB; RBP
Q8BVJ7	PDZ and LIM domain 7, isoform CRA_f	0.14522	0.36914	0.00000	0.03124	CDif; COB; D; RBP
Q8C494	Proline-rich protein 33	0.22173	0.39952	0.00012	0.04410	
F6X9B6	Nebulin (Fragment)	0.12407	0.34831	0.00000	0.02395	COB; RBP
Q8R4E4	Myozenin-3	0.16231	0.36443	0.00000	0.02945	COB
P04247	Myoglobin	0.07568	0.18258	0.00000	0.00065	CDif; RS; T
Q9JK37	Myozenin-1	0.16088	0.38551	0.00000	0.03779	COB
P11404	Fatty acid-binding protein, heart	0.09386	0.26781	0.00000	0.00641	MP; RBP; RS; T
O70250	Phosphoglycerate mutase 2	0.30921	0.31878	0.00249	0.01569	MP; RBP; RS
P14152	Malate dehydrogenase, cytoplasmic	0.39277	0.32404	0.01520	0.01702	MP

Q9DC77	Small muscular protein	0.39510	0.28952	0.01582	0.00967	
P16125	L-lactate dehydrogenase B chain	0.43554	0.25233	0.02999	0.00462	MP
P21550	Beta-enolase	0.41237	0.37481	0.02110	0.03339	MP; RS
P16015	Carbonic anhydrase 3	0.15193	0.29214	0.00000	0.01013	MP; RS
B2RQK7	Synaptopodin 2-like protein	0.11338	0.25773	0.00000	0.00519	RBP
O70400	PDZ and LIM domain protein 1	0.24498	0.32321	0.00031	0.01680	RBP; RS
Q99LX0	Protein/nucleic acid deglycase DJ-1	0.26896	0.40552	0.00075	0.04697	CC; COB; CP; DR; MP; RBP; RS; T
A2AEX8	Four and a half LIM domains 1, isoform CRA_b	0.38506	0.39510	0.01323	0.04203	CDif; D; RBP
P62897	Cytochrome c, somatic	0.43860	0.40420	0.03136	0.04634	CDe; MP; RBP; T
P05201	Aspartate aminotransferase, cytoplasmic	0.46083	0.32884	0.04239	0.01825	MP; RBP; RS
Q9R059	Four and a half LIM domains protein 3	0.30562	0.38700	0.00226	0.03843	COB
F8WJ23	Hornerin	0.01000	0.01000	0.00000	0.00000	CDif; D
Q9CQ91	NADH dehydrogenase [ubiquinone] 1 alpha subcomplex subunit 3	0.43159	0.36711	0.02830	0.03043	COB; MP; T
P48772	Cytochrome c oxidase subunit 8B, mitochondrial	0.33818	0.17756	0.00510	0.00054	MP; RS; T
A0A0G2JFH2	Microtubule-associated protein (Fragment)	0.15521	0.35100	0.00000	0.02483	
Q05793	Basement membrane-specific heparan sulfate proteoglycan core protein	0.44843	0.39324	0.03595	0.04118	CDif; COB; T
E9Q557	Desmoplakin	0.21612	0.22533	0.00010	0.00220	CDif; COB; MP; RBP; RS
P12242	Mitochondrial brown fat uncoupling protein 1		0.01000		0.00000	CDif; RBP; RS; T
P17183	Gamma-enolase	0.57904	0.38066	0.14434	0.03579	MP
Q8BU85	Methionine-R-sulfoxide reductase B3, mitochondrial	0.48309	0.32787	0.05598	0.01798	MP; RS
O08539	Myc box-dependent-interacting protein 1	0.25648	0.44033	0.00048	0.06581	CDif; COB; D; RBP; T

Q924M7	Mannose-6-phosphate isomerase	0.36390	0.56657	0.00883	0.16434	MP
O70548	Telethonin	0.46318	0.54230	0.04370	0.14207	COB; RS
Q3UGR5	Haloacid dehalogenase-like hydrolase domain-containing protein 2	0.40650	0.53220	0.01915	0.13297	MP
P70441	Na(+)/H(+) exchange regulatory cofactor NHE-RF1	0.25740	0.54289	0.00050	0.14252	CDif; COB; CH; RBP; RS; T
D3Z7P3	Glutaminase kidney isoform, mitochondrial	0.46147	0.44903	0.04279	0.07108	CC; COB; MP; RBP; RS
A0A1Y7VL34	Dystrobrevin	0.46555	0.58514	0.04504	0.18256	
O70571	[Pyruvate dehydrogenase (acetyl-transferring)] kinase isozyme 4, mitochondrial	0.43384	0.43422	0.02930	0.06221	CC; MP; RBP; RS
Q8R1G2	Carboxymethylenebutenolidase homolog	0.33898	0.48544	0.00520	0.09578	MP
Q9DAK9	14 kDa phosphohistidine phosphatase	0.25452	0.42391	0.00045	0.05639	MP; RBP
E9PYJ9	LIM domain-binding protein 3	0.20794	0.48008	0.00006	0.09176	COB
Q9ET78	Junctophilin-2	0.24600	0.44228	0.00033	0.06694	CH; D; RBP; T
Q9D0F9	Phosphoglucomutase-1	0.38447	0.41982	0.01309	0.05424	MP
E0CYV0	Protein-L-isoaspartate O-methyltransferase	0.29446	0.52056	0.00166	0.12310	MP; RBP
A0A0A6YVV8	Muscleblind-like protein 1	0.23878	0.44189	0.00024	0.06670	CDif; COB; MP; RBP
P70296	Phosphatidylethanolamine-binding protein 1	0.22193	0.48828	0.00012	0.09772	MP; RBP; RS
Q9CPU0	Lactoylglutathione lyase	0.36350	0.53419	0.00876	0.13490	CDif; MP; RBP
Q8BH80	Vesicle-associated membrane protein, associated protein B and C	0.41288	0.59880	0.02124	0.19632	COB; CH; RBP; RS; T
P09411	Phosphoglycerate kinase 1	0.40193	0.53619	0.01777	0.13647	CDif; MP; RBP; RS
Q920M5	Coronin-6	0.14959	0.48286	0.00000	0.09389	COB
P45591	Cofilin-2	0.32362	0.46425	0.00360	0.08091	COB; CH; RBP

D3YZD8	Mth938 domain-containing protein	0.19845	0.41754	0.00004	0.05306	RBP
A0A1B0GSX6	PDZ and LIM domain 3, isoform CRA_a	0.22707	0.46751	0.00015	0.08307	COB
P70670	Nascent polypeptide-associated complex subunit alpha, muscle-specific form	0.22681	0.44504	0.00015	0.06861	CCM; MP; RBP; T
Q8CI70	Leucine-rich repeat-containing protein 20	0.46041	0.58962	0.04216	0.18710	
P61971	Nuclear transport factor 2	0.27270	0.58140	0.00085	0.17883	COB; RBP; T
K3W4S6	Glycogenin	0.39277	0.52966	0.01519	0.13087	
Q9JIF9	Myotilin	0.44228	0.52219	0.03304	0.12446	
P56375	Acylphosphatase-2	0.29507	0.49237	0.00168	0.10093	
Q70IV5	Synemin	0.36684	0.45351	0.00937	0.07382	COB
P09470	Angiotensin-converting enzyme	0.31066	0.43346	0.00259	0.06174	CP; MP; RBP; RS; T
Q64691	Calpain-3	0.35791	0.49020	0.00783	0.09928	CDe; COB; MP; RBP; RS
A0A0G2JEX1	Nexilin	0.46795	0.42735	0.04642	0.05834	RBP
Q62446	Peptidyl-prolyl cis-trans isomerase FKBP3	0.21829	0.50327	0.00010	0.10913	MP
Q8R2H9	Phosphoethanolamine/phosphocholine phosphatase	0.36657	0.52715	0.00932	0.12865	MP; RBP
Q9JJV2	Profilin-2	0.40733	0.56883	0.01942	0.16667	COB; RBP
P68037	Ubiquitin-conjugating enzyme E2 L3	0.31756	0.58173	0.00310	0.17923	CP; MP; RBP; RS
O88492	Perilipin-4	0.31797	0.47393	0.00313	0.08747	
P17751	Triosephosphate isomerase	0.44170	0.42644	0.03275	0.05786	D; MP
E9QQ96	Obscurin	0.28645	0.43048	0.00131	0.06002	CDif; D; MP; RBP
E9PZF0	Nucleoside diphosphate kinase	0.46425	0.59172	0.04439	0.18917	MP; RBP
Q791T5	Mitochondrial carrier homolog 1	0.01000	0.48356	0.00000	0.09429	CDe; MP; RBP; T

Q80UY1	Carnosine N-methyltransferase	0.43783	0.46232	0.03102	0.07959	MP
S4R1W8	Glyceraldehyde-3-phosphate dehydrogenase (Fragment)	0.40783	0.45620	0.01962	0.07557	MP
O35215	D-dopachrome decarboxylase	0.40096	0.56850	0.01747	0.16621	MP; RBP
Q9D967	Magnesium-dependent phosphatase 1	0.31656	0.56085	0.00302	0.15890	MP
Q99MR9	Protein phosphatase 1 regulatory subunit 3A	0.46970	0.49727	0.04755	0.10457	MP
P61082	NEDD8-conjugating enzyme Ubc12	0.46992	0.53619	0.04759	0.13667	MP; RBP
Q8BZJ7	DCN1-like protein 2	0.35920	0.57438	0.00803	0.17183	MP; RBP
P29595	NEDD8	0.28498	0.53079	0.00125	0.13184	MP; RBP; RS
Q9DBB8	Trans-1,2-dihydrobenzene-1,2-diol dehydrogenase	0.36996	0.58275	0.00997	0.18018	MP
P59017	Bcl-2-like protein 13	0.29709	0.46019	0.00178	0.07824	CDe; RBP
P19246	Neurofilament heavy polypeptide	0.42626	0.41511	0.02614	0.05179	COB; RBP; RS
P10630	Eukaryotic initiation factor 4A-II	0.44072	0.54855	0.03230	0.14748	MP; RBP
Q6IFZ6	Keratin, type II cytoskeletal 1b	0.33944	0.57837	0.00524	0.17565	
Q8CI12	Smoothelin-like protein 2	0.30760	0.56433	0.00239	0.16236	
P47199	Quinone oxidoreductase	0.45620	0.53562	0.03995	0.13605	COB; MP
Q5DTJ9	Myopalladin	0.43197	0.49652	0.02846	0.10393	COB; RBP
P24472	Glutathione S-transferase A4	0.45005	0.52138	0.04485	0.10559	MP; RS
Q9D1G1	Ras-related protein Rab-1B	0.43725	0.58377	0.03079	0.18118	COB; MP; RBP; RS; T
Q9CQX8	28S ribosomal protein S36, mitochondrial	0.46838	0.43995	0.04676	0.06553	
Q9WVQ5	Methylthioribulose-1-phosphate dehydratase	0.47148	0.56689	0.04857	0.16470	CDe; COB; MP; RBP
Q8BLF1	Neutral cholesterol ester hydrolase 1	0.01000		0.00000		MP; RBP; RS

Q02566	Myosin-6	0.68166	0.05444	0.29197	0.00000	COB; CCM; MP; RBP; RS
P15864	Histone H1.2	0.83752	0.01000	0.58964	0.00000	COB; MP; RBP
Q9JHH6	Carboxypeptidase B2	0.99701	0.30030	0.91619	0.01167	CH; C; MP; RBP; RS
P02463	Collagen alpha-1(IV) chain	0.62422	0.01000	0.20297	0.00000	CDif; COB; RBP; RS
Q3U741	DEAD (Asp-Glu-Ala-Asp) box polypeptide 17, isoform CRA_a	0.94429	0.11036	0.80883	0.00002	CDif; COB; DR; MP; RBP; RS
O70310	Glycylpeptide N-tetradecanoyltransferase 1	0.01000	1.45560	0.00000	0.89188	MP
Q9JKB3	Y-box-binding protein 3	0.24988	0.73099	0.00038	0.34941	MP; RBP; RS
P56380	Bis(5'-nucleosyl)-tetraphosphatase [asymmetrical]	0.24149	0.67797	0.00027	0.28447	CDe
Q9JK92	Heat shock protein beta-8	0.30788	0.83472	0.00240	0.48428	
Q4VWZ5	Acyl-CoA-binding protein	0.24278	0.88968	0.00029	0.55767	CP; DR; MP; RBP; RS; T
D3Z7Q5	Programmed cell death protein 5	0.34734	0.68634	0.00625	0.29434	CDe; RBP; RS
P70349	Histidine triad nucleotide-binding protein 1	0.44883	0.71174	0.03617	0.32534	CDe; MP; RBP; RS
Q8R2G4	Ecto-ADP-ribosyltransferase 3	0.45620	0.78003	0.03997	0.41208	MP
A2AE89	Glutathione S-transferase Mu 1	0.47237	0.68587	0.04913	0.29368	MP; RS
Q9Z2Y3	Homer protein homolog 1	0.42159	0.60790	0.02438	0.20583	MP; RBP; RS
O08911	Mitogen-activated protein kinase 12	0.45351	0.64935	0.03856	0.25136	CDif; MP; RBP; RS
P26043	Radixin	0.46339	0.68493	0.04386	0.29264	COB; RBP; RS
Q8C605	ATP-dependent 6-phosphofructokinase	0.20678	0.68493	0.00006	0.29272	COB; MP
G5E8T9	Hydroxyacyl glutathione hydrolase	0.41754	0.61690	0.02286	0.21562	MP
P54923	[Protein ADP-ribosylarginine] hydrolase	0.36536	0.85179	0.00910	0.50701	MP; RBP; T
Q9CQH7	Transcription factor BTF3 homolog 4	0.39494	0.93809	0.01577	0.62296	

Q9CZ44	NSFL1 cofactor p47	0.38183	0.76336	0.01247	0.38999	COB; MP; RBP
Q9JMG1	Endothelial differentiation-related factor 1	0.37133	0.80451	0.01024	0.44407	CDif; D; MP; RBP
Q9D1X0	Nucleolar protein 3	0.43611	0.65703	0.03022	0.26018	CDe; CDif; COB; CP; CH; MP; RBP; RS; T
P54726	UV excision repair protein RAD23 homolog A	0.46404	0.68729	0.04418	0.29553	MP; RBP; RS
Q8BVI4	Dihydropteridine reductase	0.42790	0.60569	0.02678	0.20341	MP; RS
Q8BFS6	Serine/threonine-protein phosphatase CPPED1	0.37355	0.72939	0.01070	0.34688	MP
Q66JS6	Eukaryotic translation initiation factor 3 subunit J-B	0.26991	0.87260	0.00077	0.53481	MP
Q9JKB1	Ubiquitin carboxyl-terminal hydrolase isozyme L3	0.39984	0.81103	0.01717	0.45261	MP; RBP; RS
Q91VH6	Protein MEMO1	0.47148	0.91491	0.04856	0.59196	RBP
P48025	Tyrosine-protein kinase SYK	0.34106	0.62735	0.00543	0.22690	CCM; DR; MP; RBP; RS; T
A0A140T8P5	Immunoglobulin kappa chain variable 8-24 (Fragment)	0.01000	1.09170	0.00000	0.76184	
P06800	Receptor-type tyrosine-protein phosphatase C	0.40700	0.75700	0.01934	0.38237	
P56695	Wolframin	0.01000	1.11235	0.00000	0.74861	CH; MP; RBP; RS
Q64152	Transcription factor BTF3	0.43840	1.48810	0.03129	0.73998	MP; RBP; T
Q9CY58	Plasminogen activator inhibitor 1 RNA-binding protein	0.39872	0.91827	0.01685	0.59646	RBP
P63028	Translationally-controlled tumor protein	0.35298	1.19474	0.00705	0.95205	CDif; RBP
G3X8T7	Sodium channel protein	0.32289	0.91659	0.00355	0.59441	RBP; T
Q8BHG2	UPF0587 protein C1orf123 homolog	0.35549	0.82576	0.00745	0.47219	
Q8BGD9	Eukaryotic translation initiation factor 4B	0.38715	0.97847	0.01375	0.67650	COB; MP
P60670	Nuclear protein localization protein 4 homolog	0.35423	1.10742	0.00872	0.75413	COB; MP; RBP; T

Q60737	Casein kinase II subunit alpha	0.46620	0.81633	0.04542	0.45958	CDe; MP; RBP; RS
P35385	Heat shock protein beta-7	0.34771	0.86806	0.00630	0.52846	RS

Supplementary Table 2. Quantitative comparison of proteins differentially expressed after CACs treatment on days 2 and 4 (SE *vs* SC). The table includes (from left to right) Uniprot accession number, protein description, abundance ratio on days 2 and 4 (considering up-regulated ratio>1.5 and down-regulated ratio<0.6), P-value at days 2 and 4 (considering P-value<0.05 as differentially significant) and biological processes in which proteins are involved: Response to stimulus (RS), cell organization and biogenesis (COB), metabolic process (MP), regulation of biological process (RBP), transport (T), cell differentiation (CDif), cell death (CDe), cellular homeostasis (CH), coagulation (C), defense response (DR), cellular component movement (CCM), development (D), cell proliferation (CP), cell communication (CC), cell growth (CG) and cell division (CDiv).

		Abundance ratio		P-value		
**Accession**	**Description**	**SE2/SC2**	**SE4/SC4**	**SE2/SC2**	**SE4/SC4**	**Biological Process**
P02463	Collagen alpha-1(IV) chain	2.42300	100.00000	0.00001	0.00000	CDif; COB; RBP; RS
Q9Z331	Keratin, type II cytoskeletal 6B	4.24500	100.00000	0.00000	0.00000	COB; MP
P02535	Keratin, type I cytoskeletal 10	1.58000	2.31500	0.00148	0.00002	CDif; COB; RS
Q2VIS4	Filaggrin-2	100.00000	100.00000	0.00000	0.00000	
P17183	Gamma-enolase	1.85000	1.59100	0.00000	0.02215	MP
F8WJ23	Hornerin	100.00000	100.00000	0.00000	0.00000	CDif; D
O70571	[Pyruvate dehydrogenase (acetyl-transferring)] kinase isozyme 4, mitochondrial	1.66000	1.59900	0.00015	0.01559	CC; MP; RBP; RS
Q9CR86	Calcium-regulated heat stable protein 1	1.67700	1.74800	0.00934	0.03458	RBP
Q62059	Versican core protein	1.62600	1.59600	0.00821	0.03985	CDif; CCM; RBP
Q9R0H5	Keratin, type II cytoskeletal 71	2.42800	2.61200	0.00055	0.00478	COB; MP
E9QK82	Myelin protein P0	0.56300	1.71200	0.00000	0.00350	COB; RBP
P00848	ATP synthase subunit a	0.01000		0.00000		MP; RS; T
Q9QWL7	Keratin, type I cytoskeletal 17	0.67700	1.60100	0.00489	0.01688	COB; RBP; RS
P15327	Bisphosphoglycerate mutase	1.37100	1.72800	0.02502	0.02100	MP; RBP
P14069	Protein S100-A6	0.69400	1.76500	0.00188	0.00198	T
Q8BWM3	MCG130173	0.75400	1.91000	0.04755	0.00056	RBP

O89020	Afamin	0.78100	1.65900	0.04752	0.01017	T
P50543	Protein S100-A11	0.73300	1.59500	0.01829	0.01169	RBP
P07724	Serum albumin	0.75300	1.74500	0.01593	0.00246	CC; RBP; RS; T
P04919	Band 3 anion transport protein	1.27900	1.76100	0.03773	0.00208	COB; CH; RBP; RS; T
O70456	14-3-3 protein sigma	0.66400	4.51100	0.00275	0.00000	CDif; COB; RBP; RS
Q06770	Corticosteroid-binding globulin	0.69300	1.75100	0.00281	0.00418	MP; RBP; T
Q8VCT4	Carboxylesterase 1D	0.65300	1.54800	0.00030	0.01823	CDif; COB; MP; RS
F6RT34	Myelin basic protein (Fragment)	0.61100	1.57400	0.00003	0.01423	COB; RS
P70124	Serpin B5	0.90300	1.79900	0.70771	0.04013	COB; RBP
P50446	Keratin, type II cytoskeletal 6A	1.00400	3.13400	0.93924	0.00002	COB; DR; MP; RBP; RS
P05367	Serum amyloid A-2 protein	0.77500	2.00600	0.05508	0.02623	CCM; DR; RS
P61759	Prefoldin subunit 3	1.24000	1.94900	0.24388	0.01464	COB; MP
P30355	Arachidonate 5-lipoxygenase-activating protein	0.83300	1.94900	0.23455	0.00244	COB; MP; RBP; RS
Q9Z2A7	Diacylglycerol O-acyltransferase 1	0.86500	2.71700	0.64342	0.00460	COB; MP; RBP; T
P12242	Mitochondrial brown fat uncoupling protein 1	0.72100	100.00000	0.09335	0.00000	CDif; RBP; RS; T
Q91X72	Hemopexin	1.16200	1.70500	0.20733	0.00375	CH; MP; RBP; T
P06728	Apolipoprotein A-IV	0.80200	1.50400	0.05991	0.02795	COB; DR; MP; RBP; RS; T
Q9JM83	Calmodulin-4	0.94000	8.30500	0.77464	0.00000	RBP; RS
Q6S9I0	Kininogen 2	1.35200	1.85700	0.18950	0.02469	CH; RBP
P20491	High affinity immunoglobulin epsilon receptor subunit gamma	1.16800	2.06700	0.47388	0.00430	CDif; COB; CCM; DR; MP; RBP; RS; T
Q497J0	MCG130175, isoform CRA_b	0.99400	3.80600	0.84093	0.00000	RBP

Q9DCM0	Persulfide dioxygenase ETHE1, mitochondrial	1.30400	2.81700	0.19691	0.00040	MP
B2RV77	MCG130182, isoform CRA_a	1.19400	2.32300	0.27351	0.00001	RBP
Q3UP87	Neutrophil elastase	0.94700	1.57500	0.61237	0.02421	CCM; DR; MP; RBP; RS; T
Q80SZ7	Guanine nucleotide-binding protein G(I)/G(S)/G(O) subunit gamma-5	0.96900	1.74800	0.77747	0.02401	RBP; RS
Q8BND5	Sulfhydryl oxidase 1	0.89700	1.51700	0.33875	0.03474	CH; MP; RBP
Q9QXC1	Fetuin-B	0.93900	1.51600	0.53847	0.03263	RBP
P59999	Actin-related protein 2/3 complex subunit 4	0.84100	1.64300	0.15613	0.01027	COB; RBP
G3X8Q5	Ceruloplasmin	0.99300	1.52900	0.94752	0.02198	CH; MP; RS; T
Q91XL1	Leucine-rich HEV glycoprotein	0.92600	2.37500	0.48944	0.00000	CDif; RBP
P29699	Alpha-2-HS-glycoprotein	1.12200	1.51400	0.33333	0.02546	COB; DR; RBP; RS
O35744	Chitinase-like protein 3	1.05000	1.79800	0.68664	0.00140	DR; MP; RS
Q91VW3	SH3 domain-binding glutamic acid-rich-like protein 3	0.84900	1.57200	0.15620	0.01595	CH; MP; RBP
P14847	C-reactive protein	0.99600	1.85900	0.90981	0.01567	COB; DR; MP; RBP; RS
P01867	Ig gamma-2B chain C region	0.90900	1.67800	0.53429	0.03820	COB; DR; MP; RBP; RS
P02088	Hemoglobin subunit beta-1	0.92600	1.81500	0.51257	0.00117	RBP; T
P02089	Hemoglobin subunit beta-2	0.95200	1.76300	0.66878	0.00203	RBP; RS; T
P01942	Hemoglobin subunit alpha	1.09000	2.13100	0.46999	0.00003	RS; T
A0A0A6YW77	Neutrophil gelatinase-associated lipocalin (Fragment)	0.93200	1.76800	0.54835	0.00148	DR; RBP; RS; T
Q8R2S8	CD177 antigen	1.06800	2.05800	0.74658	0.00012	COB; CCM; RBP
P43883	Perilipin-2	1.04900	1.63600	0.78890	0.01142	RS; T
A2APM2	CD44 antigen	1.10000	1.70600	0.60382	0.00532	COB; CCM; MP; RBP; RS

P00920	Carbonic anhydrase 2	1.13600	1.80800	0.28447	0.00126	CH; MP; RBP; RS; T
O88545	COP9 signalosome complex subunit 6	1.02100	1.78200	0.98301	0.00187	MP
Q9CRB1	Galectin	1.05600	6.45100	0.82782	0.00000	CDe
P13634	Carbonic anhydrase 1	0.92000	1.98200	0.47091	0.00029	MP
P83870	PHD finger-like domain-containing protein 5A	1.21300	1.55200	0.10361	0.01753	MP; RBP
P28293	Cathepsin G	0.99000	1.69400	0.84080	0.00575	DR; MP; RBP; RS
A0A0R4IZY6	Myeloblastin	0.79700	2.62900	0.11427	0.00007	CDif; COB; CCM; MP; RBP; T
P17047	Lysosome-associated membrane glycoprotein 2	0.85000	1.57400	0.23247	0.02114	CC; COB; CH; MP; RBP; RS; T
Q6ZWY8	Thymosin beta-10	0.62800	2.39200	0.05767	0.00040	COB
P03975	IgE-binding protein	1.16100	2.27100	0.42704	0.00113	MP
P49138	MAP kinase-activated protein kinase 2	1.29900	1.68800	0.14092	0.03600	DR; MP; RBP; RS; T
P07091	Protein S100-A4	1.07000	1.74100	0.73219	0.00257	RBP
O09159	Lysosomal alpha-mannosidase	0.70000	2.03200	0.20615	0.03822	MP; RS
E9Q557	Desmoplakin	1.05600	5.11000	0.74778	0.00000	CDif; COB; MP; RBP; RS
Q02257	Junction plakoglobin	1.29700	2.57700	0.27079	0.00642	COB; CCM; RBP; RS
Q05816	Fatty acid-binding protein, epidermal	1.15400	1.52300	0.35741	0.03036	MP; T
P24549	Retinal dehydrogenase 1	0.96700	1.57800	0.71373	0.02033	COB; MP; RBP; RS
Q8C7X2	ER membrane protein complex subunit 1	0.83200	1.53200	0.16873	0.03039	MP
P60122	RuvB-like 1	1.03500	1.61200	0.92025	0.03308	CDiv; COB; MP; RBP; RS
P30416	Peptidyl-prolyl cis-trans isomerase FKBP4	0.92300	1.60500	0.50349	0.02656	COB; MP; RBP; RS; T
P51863	V-type proton ATPase subunit d 1	0.96700	1.70600	0.78710	0.01788	COB; CH; RS; T
P97300	Neuroplastin	0.85700	1.61300	0.25350	0.01924	COB; CH; RBP; RS

Q8R1V4	Transmembrane emp24 domain-containing protein 4	0.88400	1.77700	0.41669	0.03410	RBP; RS; T
A0A0N4SW28	Guanine nucleotide-binding protein subunit gamma	1.01300	1.89100	0.96585	0.01187	RBP; RS
P01831	Thy-1 membrane glycoprotein	1.04000	1.88900	0.90517	0.00356	CC; COB; MP; RBP; RS
Q9QZ25	Vascular non-inflammatory molecule 3	3.60100	0.26100	0.00000	0.00001	MP
A0A140T8P5	Immunoglobulin kappa chain variable 8-24 (Fragment)	100.00000	0.01000	0.00000	0.00000	
P51667	Myosin regulatory light chain 2, ventricular/cardiac muscle isoform	1.58900	0.53000	0.00009	0.00029	COB; RBP
G3X8T7	Sodium channel protein	3.32400	0.01000	0.00000	0.00000	RBP; T
P32848	Parvalbumin alpha	1.95100	0.36900	0.00000	0.00000	CH
Q9CRA2	PDZ and LIM domain protein 5	1.80600	0.35700	0.00000	0.00000	CG; RBP
Q8BVJ7	PDZ and LIM domain 7, isoform CRA_f	1.67500	0.42200	0.00001	0.00000	CDif; COB; D; RBP
Q8VC88	Grancalcin	100.00000	0.53000	0.00000	0.02241	MP
Q91Z83	Myosin-7	1.74600	0.58900	0.00000	0.00238	COB; CCM; MP; RBP; RS
Q9JJW5	Myozenin-2	1.92200	0.32800	0.00000	0.00000	COB
P56695	Wolframin	100.00000	0.01000	0.00000	0.00000	CH; MP; RBP; RS
P50462	Cysteine and glycine-rich protein 3	1.71800	0.54800	0.00028	0.00698	CDif; COB; CH; D; MP; RBP; RS
A0A0A6YWR2	Ig gamma-1 chain C region secreted form (Fragment)	2.98300	0.53400	0.00000	0.00055	
O88346	Troponin T, slow skeletal muscle	1.66800	0.38200	0.00002	0.00000	RBP
Q791T5	Mitochondrial carrier homolog 1	100.00000	0.01000	0.00000	0.00000	CDe; MP; RBP; T
Q9CQX2	Cytochrome b5 type B	1.81500	0.34200	0.00358	0.00025	MP; RBP; T
P09542	Myosin light chain 3	1.90100	0.49800	0.00000	0.00007	RBP

P04247	Myoglobin	1.90500	0.26100	0.00000	0.00000	CDif; RS; T
Q9JK37	Myozenin-1	1.50900	0.31600	0.00051	0.00000	COB
P11404	Fatty acid-binding protein, heart	1.61000	0.52800	0.00006	0.00027	MP; RBP; RS; T
B2RQK7	Synaptopodin 2-like protein	1.73000	0.42900	0.00013	0.00000	RBP
A0A0A6YVV8	Muscleblind-like protein 1	3.54000	0.58300	0.00000	0.01504	CDif; COB; MP; RBP
Q920M5	Coronin-6	2.45400	0.51500	0.00003	0.01150	COB
Q4VWZ5	Acyl-CoA-binding protein	1.75300	0.48400	0.00002	0.00002	CP; DR; MP; RBP; RS; T
Q62446	Peptidyl-prolyl cis-trans isomerase FKBP3	1.61300	0.51500	0.00121	0.00015	MP
Q9R059	Four and a half LIM domains protein 3	1.54600	0.59900	0.00359	0.00518	COB
P59017	Bcl-2-like protein 13	1.67700	0.01000	0.03834	0.00000	CDe; RBP
P35385	Heat shock protein beta-7	1.57100	0.31400	0.00140	0.00000	RS
B1AQ77	Keratin 15, isoform CRA_a	1.54900	0.27500	0.04676	0.00000	
Q9WV06	Ankyrin repeat domain-containing protein 2	1.75900	0.50700	0.00000	0.00009	CDif; RBP; RS
Q9ES74	Serine/threonine-protein kinase Nek7	3.82200	0.44300	0.00000	0.00228	MP; RBP
Q60866	Phosphotriesterase-related protein	1.52200	0.32300	0.08111	0.00049	CDif; MP
O35969	Guanidinoacetate N-methyltransferase		0.52200		0.04585	MP; RBP
Q3TTY5	Keratin, type II cytoskeletal 2 epidermal	2.63300	0.65000	0.00000	0.01705	COB; CP; CCM; MP
P28653	Biglycan	1.64200	0.69000	0.00004	0.02111	MP; RBP; RS
Q9WUZ5	Troponin I, slow skeletal muscle	1.83800	0.62300	0.00000	0.00422	RBP
P63028	Translationally-controlled tumor protein	1.53800	0.67300	0.00181	0.01388	CDif; RBP
Q9JK92	Heat shock protein beta-8	1.83100	0.66000	0.00018	0.02270	
P68037	Ubiquitin-conjugating enzyme E2 L3	1.60900	0.63100	0.00159	0.00756	CP; MP; RBP; RS

Q6P5H2	Nestin	2.07600	0.77700	0.00421	0.30196	COB; CP; D; RBP
Q8BJY1	26S proteasome non-ATPase regulatory subunit 5	100.00000	1.39200	0.00000	0.46883	COB
A0A0B4J1H7	Immunoglobulin kappa variable 1-135 (Fragment)	2.02200	0.87600	0.00101	0.54135	RS
O70310	Glycylpeptide N-tetradecanoyltransferase 1	100.00000	0.68500	0.00000	0.22778	MP
Q6PFA2	Clathrin light chain	2.03100	0.92700	0.00115	0.60151	CDiv; COB; T
P61082	NEDD8-conjugating enzyme Ubc12	1.80900	0.97600	0.00007	0.76849	MP; RBP
O88492	Perilipin-4	1.50800	0.79000	0.00104	0.12686	
P62983	Ubiquitin-40S ribosomal protein S27a	1.64200	0.74400	0.00003	0.08437	MP
D3YYD0	Olfactomedin-4	2.75500	0.73200	0.00000	0.17490	COB; RBP
Q64691	Calpain-3	1.72000	0.76700	0.02519	0.38754	CDe; COB; MP; RBP; RS
Q66JS6	Eukaryotic translation initiation factor 3 subunit J-B	1.73000	0.69000	0.02636	0.14259	MP
E9Q3E2	Synaptopodin	2.10000	1.09600	0.00000	0.84477	COB; RBP; RS
Q00897	Alpha-1-antitrypsin 1-4	2.04000	1.02500	0.00000	0.96924	RBP; RS
P01865	Ig gamma-2A chain C region, membrane-bound form	9.85600	0.98100	0.00000	0.81596	COB; DR; MP; RBP; RS; T
Q62148	Retinal dehydrogenase 2	2.04600	1.28100	0.00619	0.59381	CDif; MP; RBP; RS
A0A1Y7VL34	Dystrobrevin	1.61200	1.21700	0.01957	0.54620	
A0A0G2JEK2	Cysteine-rich protein 1	1.89300	1.23700	0.00010	0.39243	RBP; RS
P04104	Keratin, type II cytoskeletal 1	1.92000	0.98400	0.00000	0.83229	DR; MP; RBP; RS
Q8CI43	Myosin light chain 6B	1.51400	0.71500	0.00045	0.05083	CCM
Q8BLF1	Neutral cholesterol ester hydrolase 1	100.00000	1.17300	0.00000	0.78584	MP; RBP; RS

P01837	Immunoglobulin kappa constant	2.03200	0.86000	0.00000	0.35700	
Q91Y97	Fructose-bisphosphate aldolase B	2.19200	0.83200	0.00001	0.38780	COB; MP; RBP; RS
O35114	Lysosome membrane protein 2	1.69200	1.13000	0.02692	0.76660	RBP; T
Q6IFZ6	Keratin, type II cytoskeletal 1b	1.86800	0.89300	0.00000	0.40415	
Q05BC3	Echinoderm microtubule-associated protein-like 1	1.54800	0.85200	0.01685	0.44887	CDif; COB; CP
O89079	Coatomer subunit epsilon	1.80000	0.70200	0.00181	0.16979	T
Q9D4H8	Cullin-2	2.28300	0.93200	0.00002	0.61981	MP
Q05793	Basement membrane-specific heparan sulfate proteoglycan core protein	1.52100	0.98200	0.00532	0.81884	CDif; COB; T
P16045	Galectin-1	1.55300	1.11800	0.00020	0.58839	CDe; CDif; CH; RBP; RS
Q91VH6	Protein MEMO1	1.59100	1.11700	0.03420	0.78602	RBP
P08074	Carbonyl reductase [NADPH] 2	1.94900	0.94900	0.00001	0.67116	COB; MP
Q9DBG5	Perilipin-3	1.61400	1.15300	0.00681	0.62872	T
Q80XN0	D-beta-hydroxybutyrate dehydrogenase, mitochondrial	1.56500	0.91600	0.01066	0.60033	MP
D3YUE2	Procollagen C-endopeptidase enhancer 1	1.62400	0.93400	0.01427	0.65923	MP; RBP
Q9DCS3	Enoyl-[acyl-carrier-protein] reductase, mitochondrial	1.78300	0.94900	0.00808	0.74459	MP
Q9CQW1	Synaptobrevin homolog YKT6	2.04700	1.49400	0.00235	0.17721	COB; T
Q02566	Myosin-6	0.55300	0.01000	0.00560	0.00000	COB; CCM; MP; RBP; RS
P48772	Cytochrome c oxidase subunit 8B, mitochondrial	0.59700	0.01000	0.00080	0.00000	MP; RS; T
Q9Z1F9	SUMO-activating enzyme subunit 2	0.46300	0.32300	0.00018	0.00076	MP
A2CEL0	Major urinary protein 1	0.51400	0.01000	0.00665	0.00000	CC; COB; MP; RBP; RS; T

P29595	NEDD8	0.38300	0.22700	0.00000	0.00001	MP; RBP; RS
E9PYB0	AHNAK nucleoprotein 2 (Fragment)	0.41100	0.37500	0.00000	0.00003	COB
Q9QYB1	Chloride intracellular channel protein 4	0.33200	0.01000	0.00000	0.00000	CDif; COB; CH; MP; RBP; RS; T
Q91YE8	Synaptopodin-2	1.32200	0.46500	0.01787	0.00001	COB; RBP
P11087	Collagen alpha-1(I) chain	1.34400	0.56100	0.01244	0.00094	CC; CDif; COB; MP; RBP; RS; T
O08539	Myc box-dependent-interacting protein 1	1.49600	0.57100	0.00066	0.00134	CDif; COB; D; RBP; T
E9Q8K5	Titin	1.39100	0.39000	0.01631	0.00000	MP
O70250	Phosphoglycerate mutase 2	1.48800	0.54700	0.00078	0.00056	MP; RBP; RS
P16015	Carbonic anhydrase 3	1.42200	0.56100	0.00293	0.00095	MP; RS
E9PYJ9	LIM domain-binding protein 3	1.34900	0.58500	0.01153	0.00213	COB
P70296	Phosphatidylethanolamine-binding protein 1	1.49700	0.57800	0.00065	0.00168	MP; RBP; RS
P63024	Vesicle-associated membrane protein 3	0.60700	0.57400	0.01300	0.03179	COB; RBP; T
A0A0R4J0F8	Cartilage intermediate layer protein 1	0.61000	0.50400	0.02194	0.02866	RBP
Q80Z68	Rho GTPase activating protein 4, isoform CRA_f	0.71000	0.01000	0.34644	0.00000	RBP; RS
D3Z7H8	Cartilage intermediate layer protein 2	1.13800	0.53200	0.38318	0.00256	
P09528	Ferritin heavy chain	1.03500	0.55000	0.78653	0.00065	CH; MP; RBP; RS; T
P15864	Histone H1.2	0.85500	0.52500	0.29985	0.03027	COB; MP; RBP
Q8C494	Proline-rich protein 33	1.04100	0.45400	0.89784	0.00042	
F6X9B6	Nebulin (Fragment)	1.18500	0.52600	0.30225	0.00098	COB; RBP
Q8R4E4	Myozenin-3	1.30600	0.31600	0.06978	0.00000	COB
O54824	Pro-interleukin-16	1.15700	0.49700	0.49304	0.00538	CCM; MP; RBP; RS
Q9WUK2	Eukaryotic translation initiation factor 4H	1.18800	0.47200	0.34283	0.00099	COB; MP; reproduction

A4QPC5	Chymase	0.97100	0.53400	0.78849	0.00945	COB; CH; MP; RBP; RS
P62858	40S ribosomal protein S28	0.90700	0.56400	0.43526	0.00196	COB; MP
Q8R1G2	Carboxymethylenebutenolidase homolog	1.26300	0.53200	0.09752	0.00021	MP
P56380	Bis(5'-nucleosyl)-tetraphosphatase [asymmetrical]	0.71600	0.36900	0.27119	0.00151	CDe
E9PYG5	Gamma-parvin	0.89100	0.42900	0.79468	0.02109	COB
Q924M7	Mannose-6-phosphate isomerase	1.29700	0.59800	0.10200	0.00933	MP
Q3THE2	Myosin regulatory light chain 12B	0.97100	0.42500	0.73984	0.00005	RBP
F6ULR7	Myosin regulatory light chain 2, skeletal muscle isoform (Fragment)	0.98700	0.40500	0.90597	0.00000	
Q64152	Transcription factor BTF3	0.86000	0.52200	0.50585	0.01057	MP; RBP; T
Q9CQ91	NADH dehydrogenase [ubiquinone] 1 alpha subcomplex subunit 3	0.94600	0.48000	0.63276	0.00241	COB; MP; T
A0A0R4IZY0	Thimet oligopeptidase	1.08500	0.53300	0.60500	0.00029	MP; RBP; RS
Q9CY64	Biliverdin reductase A	1.03200	0.59600	0.94822	0.02265	MP
Q9DC77	Small muscular protein	0.72800	0.52600	0.29724	0.01536	
Q9DAK9	14 kDa phosphohistidine phosphatase	1.15400	0.52800	0.37008	0.00020	MP; RBP
E0CYV0	Protein-L-isoaspartate O-methyltransferase	1.28800	0.42800	0.09465	0.00003	MP; RBP
P45591	Cofilin-2	1.09800	0.47200	0.43107	0.00002	COB; CH; RBP
D3YZD8	Mth938 domain-containing protein	1.17000	0.40400	0.29393	0.00001	RBP
A0A1B0GSX6	PDZ and LIM domain 3, isoform CRA_a	1.19600	0.57400	0.18729	0.00109	COB
P70349	Histidine triad nucleotide-binding protein 1	1.16200	0.54600	0.23106	0.00029	CDe; MP; RBP; RS
P62897	Cytochrome c, somatic	1.17300	0.56600	0.17924	0.00114	CDe; MP; RBP; T
Q8R2G4	Ecto-ADP-ribosyltransferase 3	1.18600	0.38600	0.33038	0.00010	MP

O89017	Legumain	0.98100	0.48200	0.91839	0.00016	MP; RBP; RS
P13516	Acyl-CoA desaturase 1	0.83900	0.56200	0.41325	0.02600	CDif; DR; MP; RBP; RS
Q8R2H9	Phosphoethanolamine/phosphocholine phosphatase	0.93200	0.53900	0.74897	0.02459	MP; RBP
D3YVS1	Smoothelin	1.23300	0.50900	0.07670	0.00012	
Q3TMH2	Secernin-3	1.10800	0.44100	0.61934	0.00095	MP; T
P06745	Glucose-6-phosphate isomerase	1.22100	0.56600	0.09247	0.00112	MP; RBP; RS
P24472	Glutathione S-transferase A4	0.63800	0.37800	0.17928	0.00952	MP; RS
Q9D1X0	Nucleolar protein 3	0.99700	0.40900	0.91572	0.00056	CDe; CDif; COB; CP; CH; MP; RBP; RS; T
P09541	Myosin light chain 4	0.85500	0.55000	0.32679	0.02134	RBP
Q9DBB8	Trans-1,2-dihydrobenzene-1,2-diol dehydrogenase	1.30600	0.58000	0.21642	0.02789	MP
P54823	Probable ATP-dependent RNA helicase DDX6	1.34300	0.58500	0.17844	0.03634	COB; MP; RBP
Q9JLV1	BAG family molecular chaperone regulator 3	1.25100	0.54500	0.12248	0.00056	CDe; RBP; RS
Q8CI12	Smoothelin-like protein 2	1.05100	0.47100	0.84543	0.00480	
E9Q0F0	Keratin 78	1.04800	0.47500	0.89452	0.00019	COB; CCM; MP; RBP; T
Q8QZR5	Alanine aminotransferase 1	1.30800	0.42900	0.14760	0.00141	MP
Q9CQX8	28S ribosomal protein S36, mitochondrial	1.25000	0.58900	0.15550	0.00999	
P03911	NADH-ubiquinone oxidoreductase chain 4	1.09500	0.53200	0.61784	0.00038	MP; RS; T
Q8R404	MICOS complex subunit MIC13	1.19600	0.45200	0.42379	0.01765	COB
Q64310	Surfeit locus protein 4	0.93800	0.52300	0.76927	0.01852	COB; RBP
A0A0R4J032	Complement component 9	1.05500	0.58000	0.86481	0.00325	COB; C; DR; MP; RBP; RS
Q5SWU9	Acetyl-CoA carboxylase 1	0.98800	0.59100	0.82123	0.00310	COB; MP; RS

Q9QYG0	Protein NDRG2	1.17200	0.56300	0.18060	0.00101	CDif; D; RBP; RS
P62843	40S ribosomal protein S15	0.96800	0.50800	0.73890	0.00299	CDif; COB; MP; T
Q9WTL7	Acyl-protein thioesterase 2	1.00000	0.26100	0.99750	0.00000	MP
P97355	Spermine synthase	0.85300	0.50000	0.29675	0.00337	MP
Q62426	Cystatin-B	0.59200	0.63400	0.00038	0.04774	RBP; RS
E9Q8T1	Transforming acidic coiled-coil-containing protein 2	0.01000	1.10000	0.00000	0.90907	CDif; COB; CP; CCM; RBP; T
A0A140T8T7	Collagen alpha-5(VI) chain	0.20800	0.64900	0.00000	0.24036	
P32037	Solute carrier family 2, facilitated glucose transporter member 3	0.01000	1.10800	0.00000	0.96232	T
Q08943	FACT complex subunit SSRP1	0.45000	1.27900	0.00327	0.45491	MP; RBP; RS
Q9Z0M5	Lysosomal acid lipase/cholesteryl ester hydrolase	0.01000	0.61700	0.00000	0.09232	COB; CP; DR; MP; RS
Q8VCG4	Complement component C8 gamma chain	0.01000	0.64500	0.00000	0.13176	DR; MP; RBP; RS
Q9D7S7	60S ribosomal protein L22-like 1	0.49800	1.22800	0.00961	0.63144	MP
Q3V3R1	Monofunctional C1-tetrahydrofolate synthase, mitochondrial	0.42300	1.17200	0.00001	0.59182	MP
Q99L45	Eukaryotic translation initiation factor 2 subunit 2	0.47800	0.93400	0.00288	0.60075	CP; MP
O55103	Periaxin	0.56700	1.36800	0.00000	0.10601	COB; RBP
A0A1L1SRX2	AMP deaminase	0.53300	1.00400	0.00359	0.81067	MP
Q9R0P9	Ubiquitin carboxyl-terminal hydrolase isozyme L1	0.57200	0.92700	0.00015	0.60181	CC; COB; CP; CCM; MP; RBP; RS; T
V9GWW6	Muscular LMNA-interacting protein	0.48100	0.60400	0.00004	0.05216	
Q61462	Cytochrome b-245 light chain	0.58200	0.70500	0.00085	0.15911	COB; CH; DR; MP; RBP; RS; T
Q8BWF0	Succinate-semialdehyde dehydrogenase,	0.54400	1.46000	0.03158	0.40343	COB; MP

	mitochondrial					
P48024	Eukaryotic translation initiation factor 1	0.41000	0.67000	0.00000	0.11767	MP; RBP
P08551	Neurofilament light polypeptide	0.57000	1.02600	0.00001	0.97056	COB; CCM; RBP; RS; T
P16330	2',3'-cyclic-nucleotide 3'-phosphodiesterase	0.51900	1.11600	0.00058	0.77658	CDif; COB; MP; RS
O88531	Palmitoyl-protein thioesterase 1	0.56000	1.33700	0.00254	0.32598	COB; CH; MP; RBP; RS; T

Supplementary Table 3. Functional classification of proteins differentially expressed in ischemic non-treated mice (SC) *vs* sham mice (SH) on day 2 after femoral artery ligation surgery. Proteins classification was made by the IPA software based on biomedical literature and integrated databases. The table shows the most probably pathways or functions in which the proteins of interest are involved; the main categories, the related functions or diseases, p-value, molecules (protein names) and number of proteins per category.

Categories	Function	P-value	Molecules	Proteins
Cardiovascular disease	Vascular lesion	3.74E-08	ALOX5AP, APOA1, APOA4, APOB, APOE, CD44, CRP, CTSC, FGG, FLNA, FN1, HSPG2, LCAT, LOX, PARP1, PLG, PLIN2, PON1, PTPRC, SERPIND1	19
	Occlusion of artery	1.09E-16	ACE, ALDH5A1, ALOX5AP, APCS, APOA1, APOA4, APOB, APOE, C5 CA1, CA3, CD44, CLU, CRP, F10, F2, FGG, FN1, GCLM, HBA1/HBA2, Hrg, HSPG2, ITGAM, LCAT, LGALS3, LOX, LRRC20, MB, MTHFD1L, NCEH1, PARP1, PF4, PLG, PLIN2, PON1, RHOG, S100A9, SAA1, SERPINA1, SERPINA3, SERPINC1, SERPIND1, TKT, TTN	44
	Atherosclerosis	2.85E-13	ACE, ALDH5A1, ALOX5AP, APCS, APOA1, APOA4, APOB, APOE, C5 CA1, CA3, CD44, CLU, CRP, F2, FGG, FN1, GCLM, HBA1/HBA2, HP, HSPG2, ITGAM, LCAT, LGALS3, LRRC20, MTHFD1L, NCEH1, PARP1, PF4, PLG, PLIN2, PON1, S100A9, SAA1, SERPINC1, SERPIND1, TKT	36
Cardiovascular System, Cellular and Tissue Development	Angiogenesis	2.26E-09	ALDH1A2, ANXA1, ANXA3, APOA1, APOB, APOE, APOH, C3, C5, CAMP, CD44, CHI3L1, CYCS, DAB2, F2, FLNA, FN1, FSTL1, G6PD, GCLM, HMGB2, Hrg, HSPG2, Ighg2b, IQGAP1, ITGAM, KNG1, LGALS3, LOX, LRG1, MB, MYH9, Ngp, PARP1, PDE2A, PF4, PGK1, PLG, RDX, PHOG, SAA1, SERPINB5, SERPINC1, SERPIND1, SERPINF2, SYK, TKT, TPT1, VTN	49
	Vasculogenesis	7.15E-10	ALDH1A2, ANXA1, APOA1, APOB, APOE, APOH, C3, C5, CAMP, CD44, CHI3L1, CYCS, DAB2, F2, FLNA, FN1, FSTL1, G6PD, GCLM, HMGB2, HSPG2, Ighg2b, IQGAP1, ITGAM, KNG1, LGALS3, LOX, LRG1, MB, MYH9, PARP1, PDE2A, PF4, PLG, RDX, PHOG, SAA1, SERPINB5, SERPINC1, SERPIND1, SERPINF2, SYK, TKT, VTN	44
	Development of vasculature	1.92E-12	ACE, ALDH1A2, ANXA1, ANXA3, APOA1, APOB, APOE, APOH, C3, C5, CAMP, CD44, CFH, CHI3L1, CLU, CYCS, DAB2, F2, FGA, FLNA, FN1, FSTL1, G6PD, GCLM, HMGB2, Hrg, HSPB7, HSPG2, Ighg2b, IGHM, IQGAP1, ITGAM, KNG1, LGALS3, LOX, LRG1, MB, MPI, Mug1/Mug2, MYH9, Ngp, NOL3, PARP1, PDE2A, PF4, PGK1, PLG, Pzp, RDX, PHOG, SAA1, SERPINB5, SERPINC1, SERPIND1, SERPINF2, SYK, TKT, TPT1, UCHL3, VTN	60
	Endothelial cell delvelopment	4.38E-08	ALDH1A2, APOA1, APOE, APOH, C3, CAMP, CD44, DAB2, F2, FN1, G6PD, HMGB2, HSPG2, Ighg2b, IQGAP1, KNG1, LGALS3, LRG1, PDE2A, PF4, PLG, SAA1, SERPINC1, VTN	24

Cell death and survival	Apoptosis	1.38E-11	AAMDC, ACE, ALB, ALDH1A1, ANXA1, APIP, APOA1, APOE, B2M, BCL2L13, BIN1, C3, C5, CA3, CAMP, CAPN3, CD44, CFH, CHI3L1, CLU, CORO1A, CRP, CSNK2A1, CYCS, DAB2, DSP, F2, FCER1G, FGA, FHL1, FLNA, FN1, FSTL1, G6PD, GAPDH, GCLM, CLO1, GNG5, GPLD1, GSN, GSTM5, HBA1/HBA2, HMGB2, HSPB8, HSPG2, IGHM, ITGAM, ITIH4, KRT17, LGALS3, LGALS7/LGALS7B, LOX, LTF, MAP4, MB, MDH1, NCEH1, NOL3, NUDT2, OBSCN, PAK2, PARK7, PARP1, PDCD5, PF4, PLG, PRDX4, PTPRC, RANBP1, RDX, S100A6, S100A9, SAA1, SERPINA1, SERPINA3, SERPINB1, SERPINB5, SERPINC1, SFN, SH3BGRL3, SLC2A3, STK24, SYK, TMOD3, TNFAIP8, TPT1, TTN, TTR, UBE2M, VTN, WFS1, YBX3	92
	Necrosis	4.80E-12	ALB, ALDH1A1, ANXA1, APIP, APOA1, APOB, APOC3, APOE, B2M, C3, C5, CAMP, CD44, CFH, CHI3L1, CLU, CORO1A, CRP, CSNK2A1, CYCS, DAB2, DSP, F2, FABP3, FCER1G, FGA, FHL1, FLNA, FN1, FSTL1, G6PD, GAPDH, GCLM, GLO1, GSN, GSTM5, HLA-A, HMGB2, HRPT1, HSPB8, IGHM, ITGAM, ITIH4, KNG1, LAMP2, LBP, LGALS3, LGALS7/LGALS7B, LTF, LYZ, MAP4, MB, MDH1, Mug1/Mug2, NCEH1, NEFH, NEK7, NOL3, PAK2, PARK7, PARP1, PF4, PLG, PTPRC, Pzp, RDX, RPL35, S100A6, S100A9, SAA1, SERPINA1, SERPINA3, SERPINB5, SERPINC1, SFN, SYK, TMOD3, TNFAIP8, TNNT1, TPT1, TTN, TTR, UBE2L3, UBE2M, VTN, WFS1	85
Cellular movement	Cell movement	1.44E-19	ACTB, ACTR3, ALB, ALOX5AP, ANXA1, ANXA3, APCS, APOA1, APOB, APOE, ARHGAP4, ARPC2, ARPC3, BIN2, C3, C4A/C4B, C5, CAMP, CAP1, CD44, CFD, CFH, CHI3L1, Chil3/Chil4, CLU, CORO1A, CRP, CSNK2A1, CTSC, DAB2, DDT, DSP, DSTN, F10, F2, FCER1G, FGB, FHL1, FHL3, FLNA, FN1, FSTL1, G6PD, GAPSH, GC, GPLD1, GSN, Hbb-b1, Hbb-b2, HLA-1, HMGB2, HP, Hrg, HSPG2, Ighg2b, IGHM, IGKC, IQGAP1, ITGAM, KNG1, KRT6B, LBP, LCAT, LCP1, LGALS3, LOX, LTF, LYZ, MAP4, MAPK12, MYH9, NACA, NEXN, OBSCN, PAK2, PARP1, PDE2A, PDK4, PEBP1, PF4, PGLYRP1, PLG, PTPRC, RDX, RHOG, S100A9, SAA1, SERPINA1, SERPINA3, SERPINB1, SERPINB5, SERPINC1, SERPIND1, SERPING1, SLC9A3R1, STK24, SYK, TKT, TMOD3, TNFAIP8, TPI1, TPT1, TUBB, VTN	103
	Migration of cells	2.53E-16	ACTB, ACTR3, ALB, ALOX5AP, ANXA1, ANXA3, APCS, APOA1, APOB, APOE, ARHGAP4, ARPC2, C3, C4A/C4B, C5, CAMP, CAP1, CD44, CFD, CFH, CHI3L1, Chil3/Chil4, CLU, CORO1A, CRP, CSNK2A1, CTSC, DAB2, DDT, DSP, F10, F2, FCER1G, FGB, FHL1, FLNA, FN1, FSTL1, G6PD, GC, GPLD1, Hbb-b1, Hbb-b2, HLA-A, HP, Hrg, Ighg2b, IGHM, IGKC, IQGAP1, ITGAM, KNG1, KRT6B, LBP, LCAT, LCP1, LGALS3, LOX, LYZ, MAP4, MAPK12, MYH9, NACA, NEXN, PAK2, PARP1, PDE2A, PDK4, PF4, PGLYRP1, PLG, PTPRC, RDX, RHOG, S100A9, SAA1, SERPINA1, SERPINA3, SERPINB1, SERPINB5, SERPINC1, SERPIND1, SERPING1, SLC9A3R1, STK24, SYK, TMOD3, TNFAIP8, TPT1, VTN	90

Supplementary Table 4. Functional classification of differently expressed proteins in ischemic non-treated mice (SC) *vs* sham mice (SH) on day 4 after femoral artery ligation surgery. Proteins classification was made by the IPA software based on biomedical literature and integrated databases. The table shows the most probably pathways or functions in which the proteins of interest are involved; the main categories, the related functions or diseases, p-value, molecules (protein names) and number of proteins per category.

Categories	Function	P-value	Molecules	Proteins
Cardiovascular Disease	Vascular lesion	6,85E-13	ALOX5AP, APOA1, APOA4, APOB, APOE, CD44, CRP, CTSC, FGG, FLNA, FN1, HMOX1, HSPG2, LCAT, LOX, NCF1, PARP1, PFN1, PLG, SERPIND1, VCAN	21
	Occlusion of artery	3,86E-19	ALOX5AP, APCS, APOA1, APOA4, APOB, APOE, C5, CA3, CD14, CD44, CLU, CRP, F10, F13A1, F2, FGG, FN1, GCLM, HMOX1, Hrg, HSPG2, LCAT, LGALS3, LOX, MB, NCF1, PARP1, PF4, PFN1, PLG, PLIN2, RHOG, S100A9, SERPINA1, SERPINA3, SERPIND1, TTN, UCP1, VCAN	39
	Atherosclerosis	1,66E-14	ALOX5AP, APCS, APOA1, APOA4, APOB, APOE, C5, CA3, CD14, CD44, CLU, CRP, F13A1, F2, FGG, FN1, GCLM, HMOX1, HSPG2, LCAT, LGALS3, NCF1, PARP1, PF4, PFN1, PLG, PLIN2, S100A9, SERPIND1, UCP1, VCAN	31
	Peripheral vascular disease	5,95E-10	APOA1, APOB, APOE, APOH, B2M, CA3, CFH, COL4A1, CRP, CTSB, CTSC, DAB2, F10, F13A1, F2, FABP3, LCP1, PDLIM5, PLG, S100A9, SERPINA1, SERPINA3, SERPIND1, SLC2A3, VIM	25
Cardiovascular System, Cellular and Tissue Development	Angiogenesis	1,16E-14	ALDH1A2, ANXA1, APOA1, APOB, APOE, APOH, ARG1, C3, C5, CAMP, CD44, CHI3L1, COL4A1, CTSB, CYCS, DAB2, F2, FBLN2, FGR, FLNA, FN1, FSTL1, G6PD, GCLM, HMOX1, Hrg, HSPG2, Ighg2b, IQGAP1, KNG1, LGALS3, LOX, LRG1, MB, MYH9, NCF1, Ngp, PARP1, PDE2A, PF4, PLG, RHOG, SERPINB5, SERPIND1, SGPL1, VIM, VTN	47
	Vasculogenesis	1,40E-14	ALDH1A2, ANXA1, APOA1, APOB, APOE, APOH, ARG1, C3, C5, CAMP, CD44, CHI3L1, COL4A1, CTSB, CYCS, DAB2, F2, FLNA, FN1, FSTL1, G6PD, GCLM, HMOX1, HSPG2, Ighg2b, IQGAP1, KNG1, LGALS3, LOX, LRG1, MB, MYH9, PARP1, PDE2A, PF4, PLG, RHOG, SERPINB5, SERPIND1, SGPL1, VIM, VTN	42
	Development of vasculature	3,55E-16	ALDH1A2, ANXA1, APOA1, APOB, APOE, APOH, ARG1, C3, C5, CAMP, CD44, CFH, CHI3L1, CLU, COL4A1, CTSB, CYCS, DAB2, F2, FBLN2, FGA, FGR, FLNA, FN1, FSTL1, G6PD, GCLM, HMOX1, Hrg, HSPG2, Ighg2b, IQGAP1, KNG1, LGALS3, LOX, LRG1, MB, Mug1/Mug2, MYH9, NCF1, Ngp, PARP1, PDE2A, PF4, PLG, PLOD1, Pzp, RHOG, SERPINB5, SERPIND1, SGPL1, VIM, VTN	53
	Endothelial cell development	1,46E-11	ALDH1A2, APOA1, APOE, APOH, ARG1, C3, CAMP, CD44, COL4A1, DAB2, F2, FN1, G6PD, HMOX1, HSPG2, Ighg2b, IQGAP1, KNG1, LGALS3, LRG1, PDE2A, PF4, PLG, VTN	24
Cell Morphology, Tissue Development	Tubulation of cells	5,80E-06	ANXA1, CD44, CHI3L1, CSRP3, FLNA, G6PD, PARP1, PLG, SERPIND1, VIM	10

Cell Death and Survival	Apoptosis	2,07E-10	ALDH1A2, ANXA1, APOA1, APOE, ARG1, B2M, C3, C5, CA3, CAMP, CD14, CD44, CFH, CHI3L1, CLU, COL4A1, CRP, CTSB, CTSZ, CYCS, DAB2, DSP, F2, FCER1G, FGA, FHL1, FLNA, FN1, FSTL1, G6PD, GCLM, GSN, HIST1H1C, HMOX1, HSPG2, KRT17, LGALS3, LGMN, LOX, LTF, MAP4, MB, MDH1, MSRB3, NCF1, PARK7, PARP1, PF4, PFN1, PLG, PRDX4, S100A4, S100A6, S100A9, SERPINA1, SERPINA3, SERPINB1, SERPINB5, SGPL1, SH3BGRL3, SLC2A3, STMN1, TNFAIP8, TTN, TTR, UCP1, VTN	67
	Necrosis	1,99E-16	ALDH1A2, ANXA1, APOA1, APOB, Apoc3, APOE, B2M, C3, C5, CAMP, CD14, CD44, CFH, CHI3L1, CLU, COL4A1, CPB2, CRP, CTSB, CTSZ, CYCS, DAB2, DSP, EEF1A1, F13A1, F2, FABP3, FCER1G, FGA, FHL1, FLNA, FN1, FSTL1, G6PD, GCLM, GSN, HIST1H1C, HLA-A, HMOX1, HPRT1, LAMP2, LBP, LGALS3, LGMN, LTF, LYZ, MAP4, MB, MDH1, Mug1/Mug2, MYH6, NCF1, PABPC1, PARK7, PARP1, PF4, PFN1, PLG, Pzp, RPL3, S100A6, S100A9, SERPINA1, SERPINA3, SERPINB5, SGPL1, STMN1, TNFAIP8, TNNT1, TTN, TTR, UCP1, VTN	73
Cellular Movement	Cell movement	2,79E-26	ACTB, ALOX5AP, ANXA1, APBB1IP, APCS, APOA1, APOB, APOE, ARG1, ARHGAP4, ARPC2, ARPC3, C3, C4A/C4B, C5, CAMP, CAP1, CD14, CD44, CFD, CFH, CHI3L1, Chil3/Chil4, CLU, COL4A1, CPB2, CRP, CTSB, CTSC, CTSZ, DAB2, DSP, F10, F13A1, F2, FBLN2, FCER1G, FGB, FGR, FHL1, FHL3, FLNA, FN1, FSCN1, FSTL1, G6PD, GC, GSN, HLA-A, HMOX1, HP, Hrg, HSPG2, Ighg2b, IGKC, IQGAP1, KNG1, LBP, LCAT, LCP1, LGALS3, LGMN, LOX, LYZ, MAP4, MYH6, MYH9, NCF1, PARP1, PDE2A, PF4, PFN1, PLG, RCC2, RHOG, S100A4, S100A9, SERPINA1, SERPINA3, SERPINB1, SERPINB5, SERPIND1, SERPING1, SGPL1, STMN1, TNFAIP8, TUBB, VCAN, VIM, VTN	90
	Migration of cells	1,39E-25	ACTB, ALOX5AP, ANXA1, APBB1IP, APCS, APOA1, APOB, APOE, ARHGAP4, ARPC2, C3, C4A/C4B, C5, CAMP, CAP1, CD14, CD44, CFD, CFH, CHI3L1, Chil3/Chil4, CLU, COL4A1, CPB2, CRP, CTSB, CTSC, CTSZ, DAB2, DSP, F10, F13A1, F2, FBLN2, FCER1G, FGB, FGR, FHL1, FLNA, FN1, FSCN1, FSTL1, G6PD, GC, GSN, HLA-A, HMOX1, HP, Hrg, Ighg2b, IGKC, IQGAP1, KNG1, LBP, LCAT, LCP1, LGALS3, LGMN, LOX, LYZ, MAP4, MYH6, MYH9, NCF1, PARP1, PDE2A, PF4, PFN1, PLG, RCC2, S100A4, S100A9, SERPINA1, SERPINA3, SERPINB1, SERPINB5, SERPIND1, SERPING1, SGPL1, STMN1, TNFAIP8, VCAN, VIM, VTN	84

Supplementary Table 5. Primary antibodies used in this study.

Antibody	Reactivity	Dilution	Suplier	Reference	Used in
CD31-FITC	Human	1:25	Biolegend	303103	FC
CD34-APC	Human	1:25	Biolegend	343607	FC
CD45-PBlue	Human	1:25	Biolegend	368539	FC
CD90-PE	Human	1:25	Biolegend	328109	FC
CD73-FITC	Human	1:25	Biolegend	344015	FC
CD105-FITC	Human	1:25	Biolegend	323203	FC
CD309-PE	Human	1:25	Biolegend	359903	FC
CD133-PE	Human	1:25	Miltenyi Biotec	130-098-826	FC
CD146-PE	Human	1:25	Biolegend	361005	FC
CD14-FITC	Human	1:25	Biolegend	367115	FC
α-Actin smooth muscle	Mouse	1:500	ThermoFisher	MA5-14084	IHC
CD31	Human	1:500	Abcam	AB32457	IHC
Ly-6G	Mouse	1:500	Biolegend	127601	IHC
Apo-E	Mouse	1:1000	ThermoFisher	PA5-78803	WB

Supplementary table 6. Secondary antibodies used in this study.

Antibody	Reactivity	Dilution	Suplier	Reference	Used in
Alexa Fluor 488 anti-rabbit IgG	Rabbit	1:500	ThermoFisher	R37116	IHC
Alexa Fluor 555 anti-rat IgG	Rat	1:500	ThermoFisher	A21434	IHC
HRP anti-rabbit IgG	Rabbit	1:5000	Novus	NB160	WB

FC: Flow cytometry; IHC: Immunohistochemistry; WB: Western Blot

Additionally, for cell detection FITC-UEA-1 (1:300, Sigma L9006) and DiI-ac-LDL (Biomedical Technologies, BT-902, Stoughton, MA, USA) were used.

For IHC, the blocking reagent was 5% goat serum and 0.1% triton. Goat serum was obtained from Vector laboratories (ref. S-1000).
